# Supplementary material for: Selective activation of four quasi-equivalent C–H bonds yields N-doped graphene nanoribbons with partial corannulene motifs
Source: Nat Commun. 2022 Oct 17;13:6146. doi: 10.1038/s41467-022-33898-2 (PMC9576682; doi:10.1038/s41467-022-33898-2)
Supplement: Supplementary file 1 — Supplementary Information [file 41467_2022_33898_MOESM1_ESM.pdf]

Supplementary Information

Selective activation of four quasi-equivalent C-H bonds yields N-doped graphene nanoribbons with partial corannulene motifs

Yixuan Gao[1▲], Li Huang[1▲], Yun Cao[1▲], Marcus Richter[2▲], Jing Qi[1], Qi Zheng[1], Huan Yang[1], Ji Ma[2], Xiao Chang[1], Xiaoshuai Fu[1], Carlos-Andres Palma[1], Hongliang Lu[1], Yu-Yang Zhang[1], Zhihai Cheng[3], Xiao Lin[1], Min Ouyang[4], Xinliang Feng[*, 2, 5], Shixuan Du[*,1, 6] and Hong-Jun Gao[*,1, 6]

[1]*Institute of Physics & University of Chinese Academy of Sciences, Chinese Academy of Sciences, Beijing 100190, PR China*

[2]*Center for Advancing Electronics Dresden (cfaed) & Faculty of Chemistry and Food Chemistry, Technische Universität Dresden, D-01069 Dresden, Germany*

[3]*Department of Physics and Beijing Key Laboratory of Optoelectronic Functional Materials & Micro-nano Devices, Renmin University of China, 100872 Beijing, China*

[4]*Department of Physics, University of Maryland, College Park, MD 20742, USA*

[5]*Max Planck Institute of Microstructure Physics, Weinberg 2, Halle, 06120 Germany*

[6]*Songshan Lake Materials Laboratory, Dongguan, Guangdong 523808, PR China*

[▲]These authors contributed equally.

Corresponding Authors:

Xinliang Feng: *xinliang.feng@tu-dresden.de

Shixuan Du: *sxdu@iphy.ac.cn

Hong-Jun Gao: *hjgao@iphy.ac.cn

Contents

Supplementary Discussion

Supplementary Note 1. General Information

Supplementary Note 2. Synthesis and Characterization of Precursor (**1**)

Supplementary Note 3. High-Resolution Mass Spectroscopy

Supplementary Note 4. NMR Spectroscopy

Supplementary Note 5. Infrared Spectroscopy

Supplementary Figures

Supplementary References

■ Supplementary Discussion

Supplementary Note 1. General Information

Unless otherwise stated, the commercially available reagents and dry solvents were used without further purification. The reactions were performed using standard vacuum-line and Schlenk techniques, work and purification of all compounds were performed under air and with reagent-grade solvents.

For microwave-assisted reactions a CEM Discover-SP was used.

Column chromatography was done with silica gel (particle size 0.063-0.2 mmm from VWR) and silica coated aluminum sheets with fluorescence indicator from Merck were used for thin layer chromatography. Purification by recycling gel permeation chromatography (rGPC) was performed on JAI HPLC LC 9110 II NEXTwith fraction collector FC3310 and GPC columns 2H and 1H (connected in series). The rGPC was used with HPLC-grade chloroform at room temperature.

NMR Data were recorded on a Bruker AV-III 600 spectrometer operating at 600 MHz for 1H and 151 MHz for ^{13}C with standard Bruker puls programs at room temperature. Chemical shifts *δ* are given in ppm relative to TMS, coupling constants *J* are given in Hertz. $C_2D_2Cl_4$ (δ(^{1}H) = 5.91 ppm, δ(^{13}C) = 74.2 ppm) or C_2D_6OS (δ(^{1}H) = 2.50 ppm, δ(^{13}C) = 39.56 ppm) were used as solvent, lock and internal standard.

HR-MALDI-TOF MS spectrum were recorded on a Bruker Autoflex Speed MALDI-TOF MS (Bruker Daltonics, Bremen, Germany) with dithranol or trans-2-[3-(4-*tert*-butylphenyl)-2-methyl-2-propenylidene]malononitrile (DCTB) as the matrix. The preparation of the sample was performed in solid state.

High-Resolution Electrospray Ionization (ESI) mass spectra were recorded with an Agilent 6538 Ultra High Definition (UHD) Accurate-Mass Q-TOF LC/MC system using the positive mode.

Infrared spectra were recorded on Bruker Tensor II with a diamond ATR unit.

Supplementary Note 2. Synthesis and Characterization of Precursor (1)

The synthesis and the analytical data of 1,5-dibora-2,6-dioxa-*sym*-hydrindacene-1,5-diol (**10**) are provided in our previous work.[1]

Synthesis of 2-bromo-6-(1'-hydroxymethylphenyl))-aniline (9)

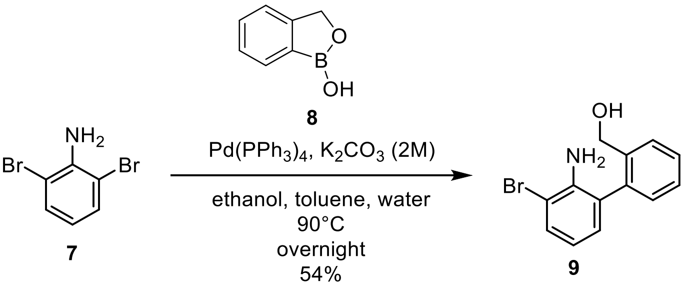

A solution of 2,6-dibromoaniline (**7**) (4 g, 16 mmol, 1.1 eq.) and 1-hydroxy-3*H*-2,1-benzoxaborole (**8**) (1.95 g, 1.45 mmol, 1.0 eq.) in a mixture of toluene (320 ml), ethanol (64 ml) and 2 M potassium carbonate solution (124 ml) was purged with argon for 30 min. After the addition of tetrakis(triphenylphosphine)palladium(0) (1.67 g. 14.5 mmol, 10 mol-%) the mixture was refluxed in an oil bath overnight at 90 °C. Afterwards, the mixture was cooled to room temperature and quenched with water. The organic layer was separated and the aqueous phase was extracted three times with diethylether. The combined organic layers were washed with brine and dried over magnesium sulfate. The solvent was removed under reduced pressure and the residue was purified by column chromatography on silica (*i*-hexane: ethyl acetate: 7:3). The crude product was purified by rGPC in chloroform to obtain 2-bromo-6-(1'-hydroxymethylphenyl))-aniline (**9**) in 54% yield.

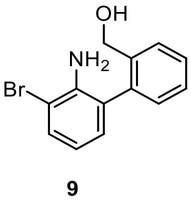

9

9: R_f = 0.4 (SiO_2; *i*-hexane/EA = 7:3). Mp = 257°C

^{1}H-NMR (600 MHz, DMSO-d$_6$): δ 7.64 – 7.59 (m, 1H), 7.45 – 7.39 (m, 2H), 7.34 (td, *J* = 7.5, 1.0 Hz, 1H), 7.11 (dd, *J* = 7.5, 1.2 Hz, 1H), 6.90 (dd, *J* = 7.4, 1.4 Hz, 1H), 6.65 – 6.56 (m, 1H), 5.07 (t, *J* = 5.3 Hz, 1H), 4.44 (s, 2H), 4.26 (ddd, *J* = 32.5, 13.7, 5.3 Hz, 2H).

^{13}C-NMR (151 MHz, DMSO-d$_6$): 142.3, 140.6, 136.1, 131.6, 129.5, 129.4, 128.0, 127.3, 127.2, 126.8, 117.8, 108.3, 60.4.

HR-MS (ESI-MS): m/z ([M+H]$^+$) = 278.0173, calcd. for $C_{13}H_{13}BrNO$: m/z = 278.0180, error = - 2.5 ppm.

IR: $\tilde{\nu}$ = 3459, 3373, 1605, 1443, 1042, 730 cm^{-1}.

Synthesis of (2',2'''-diamino-[1,1':3',1'':4'',1''':3''',1''''-quinquephenyl]-2,2'',2'''',5''-tetrayl)tetramethanol (11)

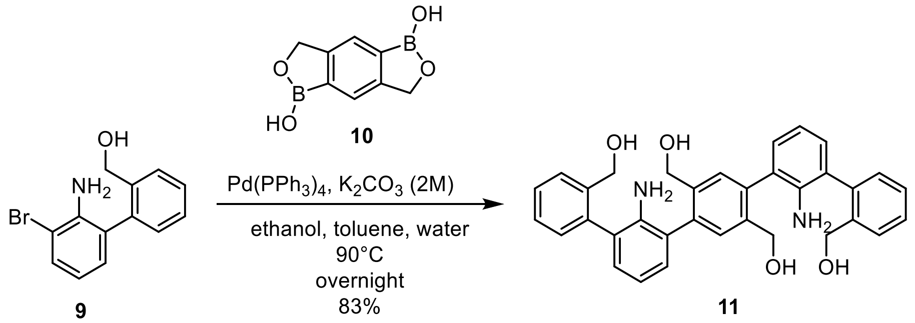

A solution of 2-bromo-6-(1'-hydroxymethylphenyl))-aniline (**9**) (3 g, 10.8 mmol, 3.0 eq.) and 1,5-dibora-2,6-dioxa-*sym*-hydrindacene-1,5-diol (**10**) (1 g, 5.3 mmol, 1.0 eq.) in a mixture of toluene (150 ml). ethanol (70 ml) and 2 M potassium carbonate solution (90 ml) was purged with argon for 30 min. After the addition of tetrakis(triphenylphosphine)palladium(0) (0.3 g, 263 mmol, 5 mol-%) the mixture was refluxed in an oil bath overnight at 90 °C. Afterwards, the mixture was cooled to room temperature and quenched with water. The organic layer was separated and the aqueous phase was extracted three times with dichloromethane. The combined organic layers were washed with brine and dried over magnesium sulfate. The solvent was removed under reduced pressure. The residue was dissolved in a minimum amount of DCM (~15 mL) and precipitated in toluene (300 ml). The precipitated solid was filtered and compound (**11**) was obtained as white solid in 83 % yield.

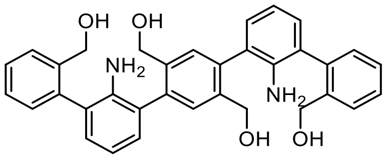

11

11: Mp = 214°C

^{1}H-NMR (600 MHz, DMSO-d$_6$): δ 7.69 – 7.57 (m, 2H), 7.48 – 7.30 (m, 6H), 7.23 – 7.12 (m, 2H), 7.03 – 6.96 (m, 2H), 6.95 – 6.86 (m, 2H), 6.83 – 6.66 (m, 2H), 5.19 – 4.89 (m, 4H), 4.60 – 4.18 (m, 8H), 3.80 (dd, *J* = 73.5, 16.1 Hz, 4H).

^{13}C-NMR (151 MHz, DMSO-d$_6$): δ 141.93, 141.91, 141.85, 140.98, 140.95, 140.73, 140.66, 139.41, 139.25, 139.19, 137.02, 136.84, 136.83, 136.70, 135.68, 129.83, 129.63, 129.22, 129.16, 129.13, 129.11, 129.00, 128.25, 128.16, 128.04, 127.60, 127.59, 127.55, 127.19, 127.02, 126.84, 125.62, 125.60, 125.58, 125.53, 125.42, 125.42, 116.83, 116.67, 116.55, 116.53, 116.42, 60.56, 60.52, 60.44, 60.37, 60.34, 60.26, 60.24.

HR-MS (ESI-MS): m/z ([M+H]$^+$] = 533.2432, calcd. for $C_{34}H_{33}N_2O_4$: m/z = 533.2440, error = - 1.5 ppm.

IR: $\tilde{\nu}$ = 3357, 3259, 1609, 1438,1039, 754 cm^{-1}.

Hydrogen bonds between amino and hydroxyl substituents hinder free rotation of the phenyl substituents. This causes isomer formation and explains the complex ^{1}H-and ^{13}C-spectra.

Synthesis of 10,19,21-trihydro-8*H*-pyrido[3,2,1-*de*]phenanthridine[2,3-*j*]isoquinolino[4,3,2-*de*]phenanthridine (1)

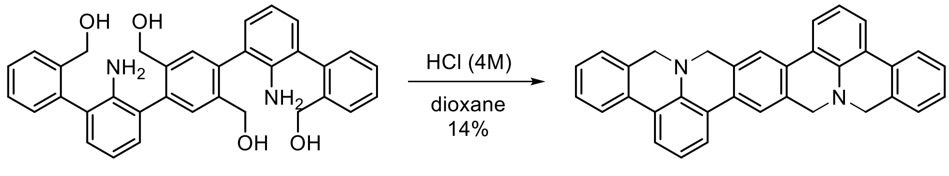

11 **1**

In a microwave tube, the intermediate (**11**) (0.1 g, 188 µmol.) was added into a stirring anhydrous hydrogen chloride solution (4 M in dioxane, 5 ml). The microwave tube was capped and placed in a microwave reactor. A dynamic mode was chosen (300 W, power max: on, activated cooling, pre-stirring: 10 seconds, temperature: 130 °C) for 90 minutes. After cooling to room temperature the reaction mixture was transferred into the glovebox. Under glovebox conditions, the precipitated solid was filtered and washed with dry methanol (50 ml). The final compound (**1**) was obtained as green/brown solid.

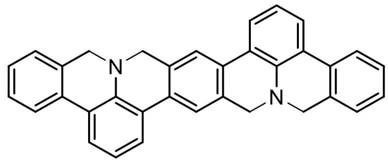

1

1: Mp = 395°C

^{1}H-NMR (600 MHz, $C_2D_2Cl_4$):) δ 7.62 – 7.53 (m, 6H), 7.36 (s, 2H), 7.27 (td, *J* = 7.8, 1.1 Hz, 2H), 7.19 (td, *J* = 7.4, 0.9 Hz, 2H), 7.13 (t, *J* = 7.5 Hz, 2H), 6.85 (t, *J* = 7.7 Hz, 2H), 4.19 (d, *J* = 13.2 Hz, 8H).

^{13}C-NMR (151 MHz, $C_2D_2Cl_4$): 143.1, 131.7, 131.3, 130.8, 130.6, 128.5, 128.0, 126.5, 123.7, 123.5, 123.0, 122.9, 122.3, 120.4, 120.3, 54.3, 54.2.

HR-MS (MALDI-ToF): m/z ($[M+-H]^+$) = 459.1847, calcd. for $C_{34}H_{23}N_2$: m/z = 459.1861, error = - 3.0 ppm.

IR: $\tilde{\nu}$ = 2764, 1421, 1267, 754 cm^{-1}.

Due to the low solubility in d_2-tetrachloroethane, chemical shifts of 13-C nuclei were derived from the HSQC or from HMBC experiments.

Supplementary Note 3. High-Resolution Mass Spectroscopy

3.1 High-Resolution Electrospray Ionization Mass Spectroscopy (HR-ESI-MS)

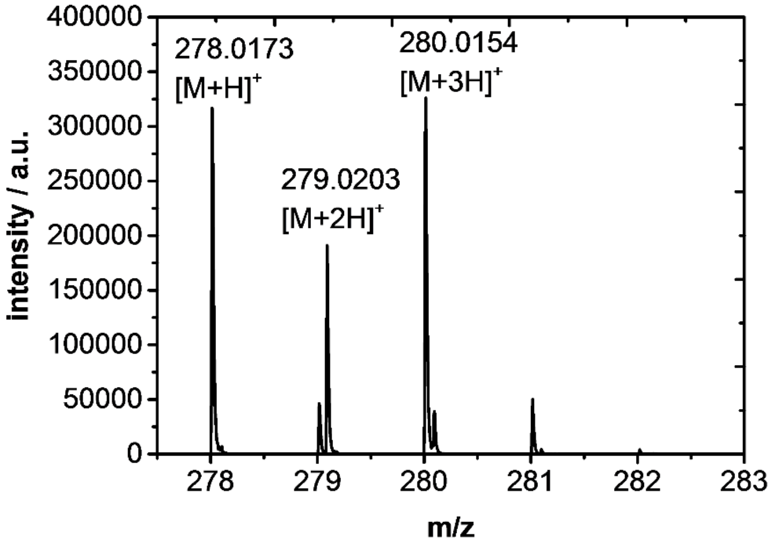

Supplementary Figure 1. HR-ESI spectrum of **9**.

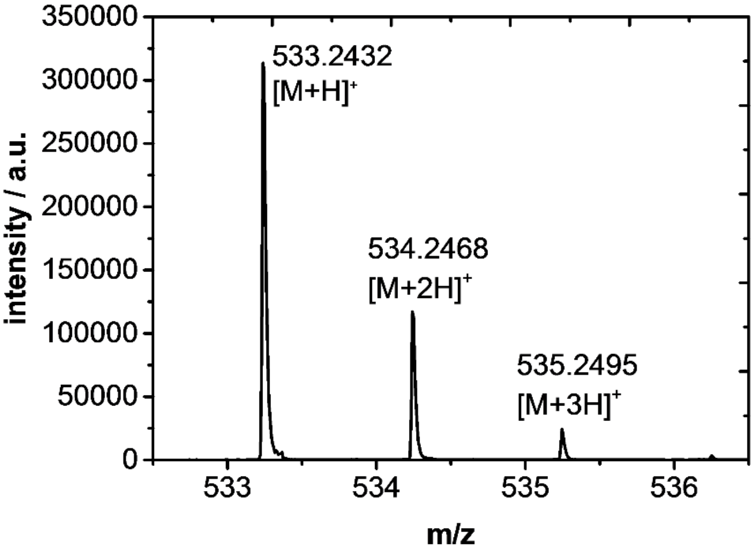

Supplementary Figure 2. HR-ESI spectrum of **11**.

3.2 High-Resolution Matrix Assisted Laser Desorption/Ionization Time of Flight Mass Spectroscopy (HR MALDI-TOF MS)

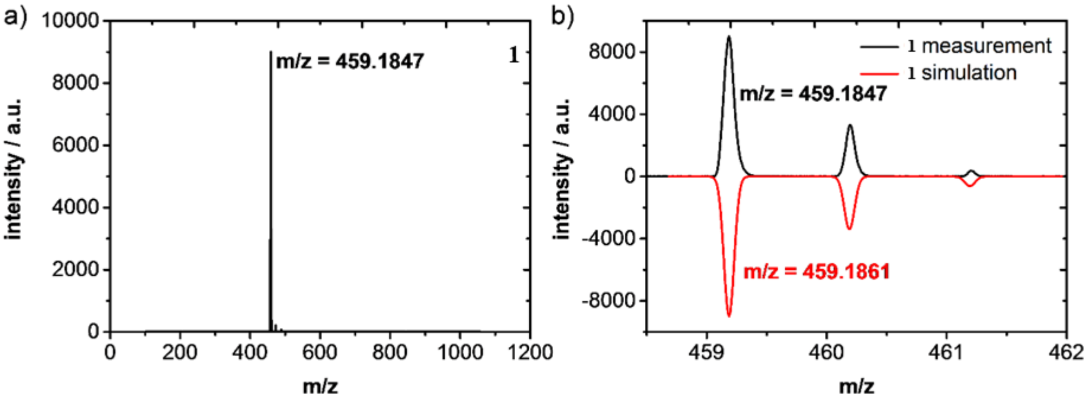

Supplementary Figure 3. a) HR MALDI-TOF mass spectrum of **1**; b) HR MALDI-TOF mass measurement of **1** (black line) is in agreement to the expected isotopic distribution pattern (red line).

Supplementary Note 4. NMR Spectroscopy

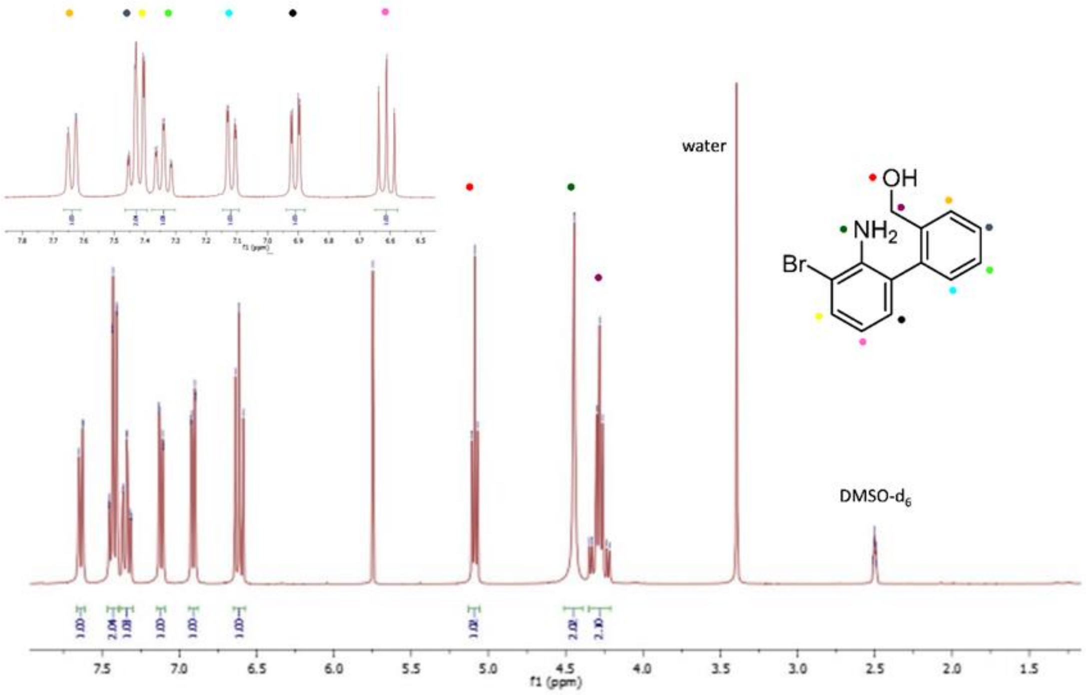

Supplementary Figure 4. ^{1}H-NMR spectrum (300 MHz) of **9** at 298 K in DMSO-d$_6$.

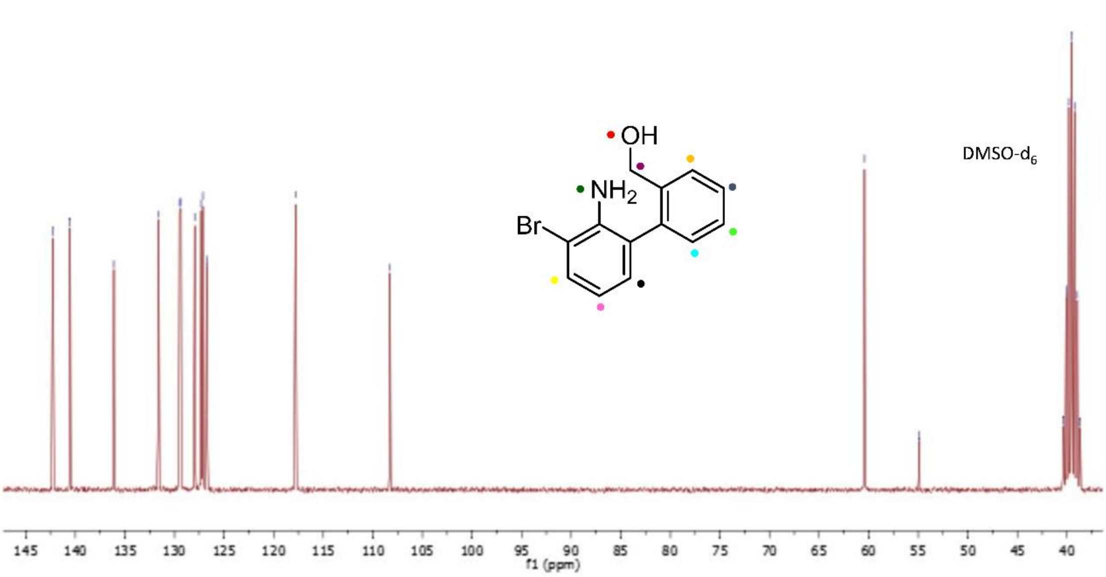

Supplementary Figure 5. ^{13}C-NMR spectrum (300 MHz) of **9** at 298 K in DMSO-d$_6$.

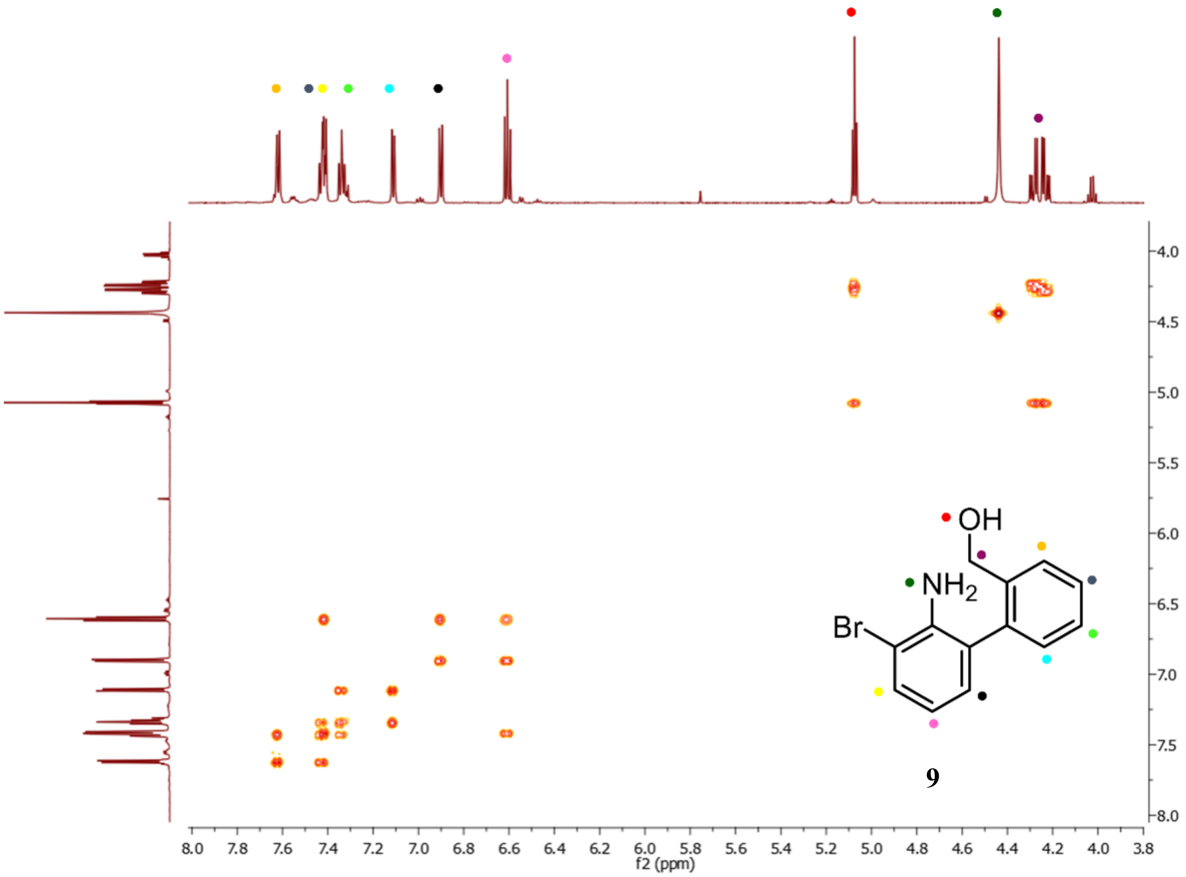

Supplementary Figure 6. COSY spectra of compound **9** at 298 K in DMSO-d_6.

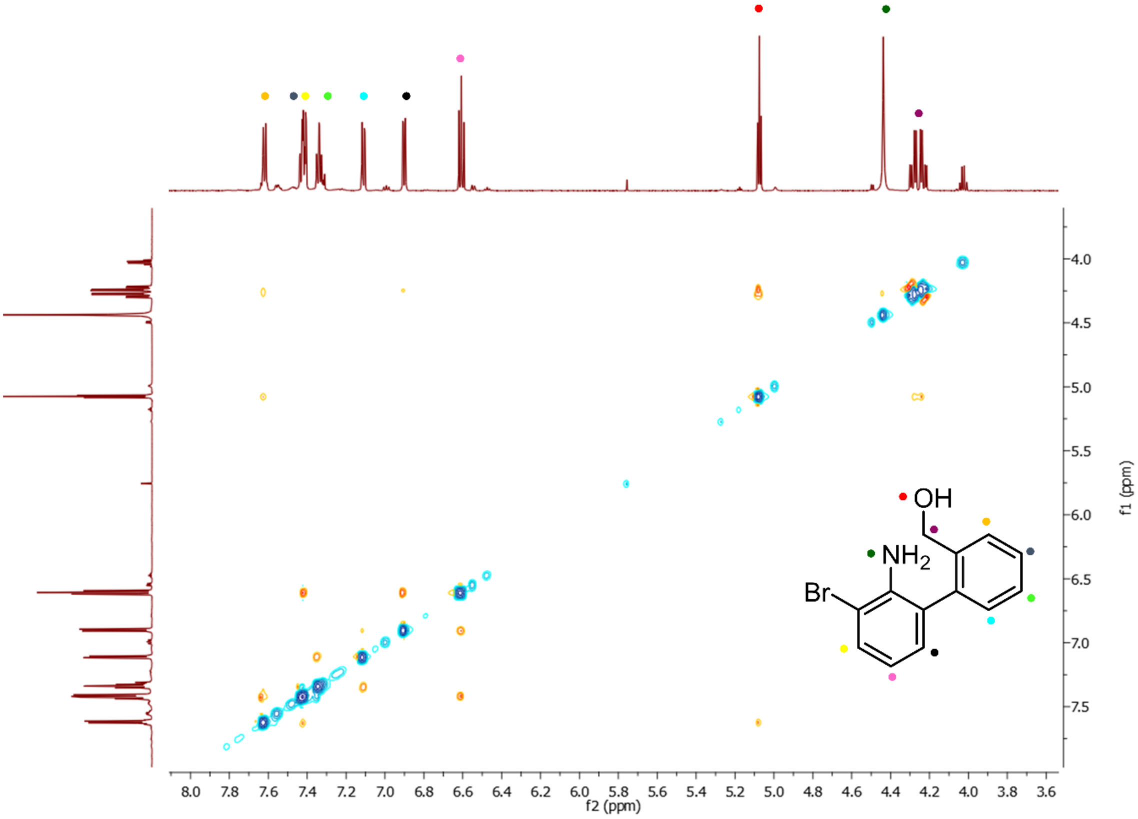

Supplementary Figure 7. NOESY spectra of compound **9** at 298 K in DMSO-d_6.

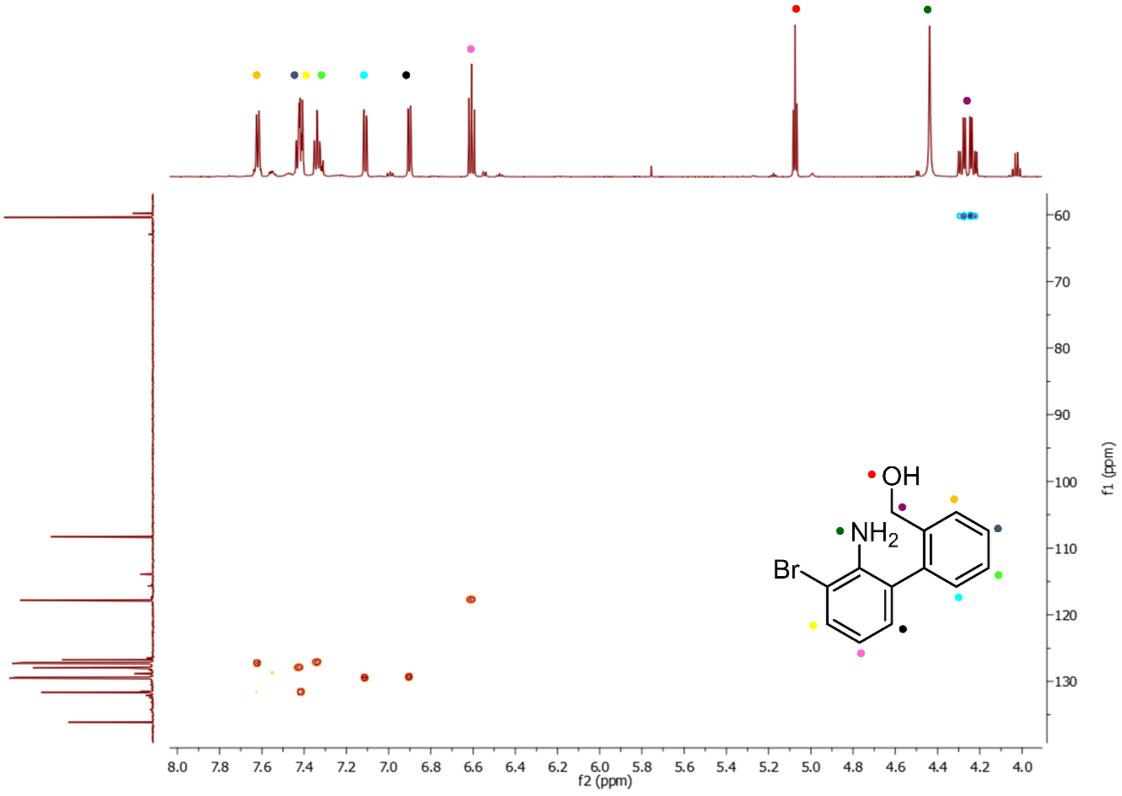

Supplementary Figure 8. HSQC spectra of compound **9** at 298 K in DMSO-d_6.

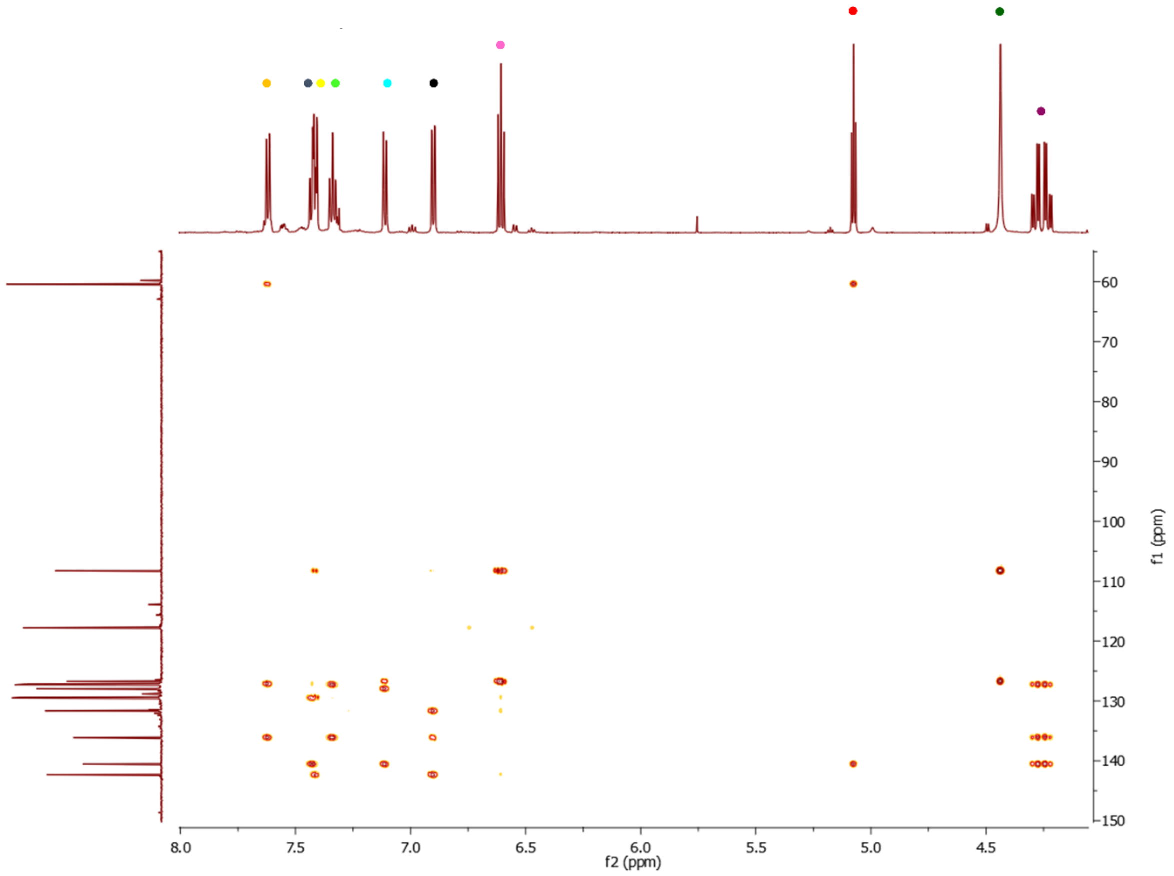

Supplementary Figure 9. HMBC spectra of compound **9** at 298 K in DMSO-d_6.

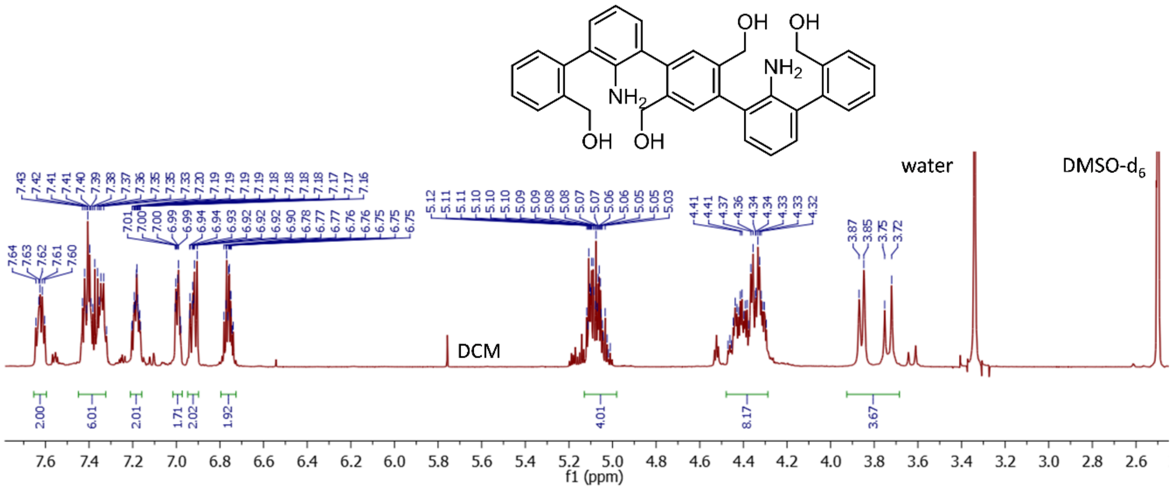

Supplementary Figure 10. ^{1}H-NMR (600 MHz) spectra of compound **11** at 298 K in DMSO-d_6.

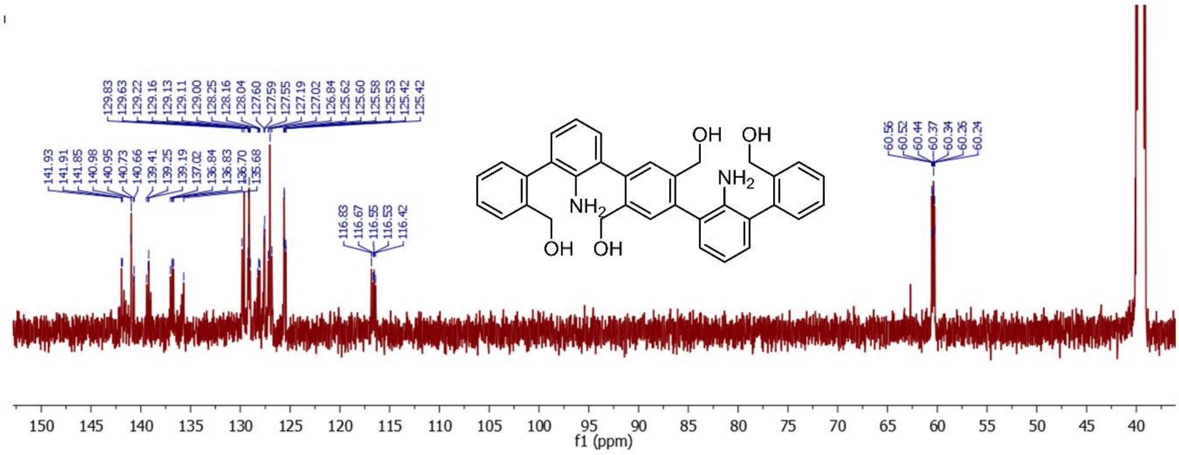

Supplementary Figure 11. ^{13}C-NMR (151 MHz) spectra of compound **11** at 298 K in DMSO-d_6.

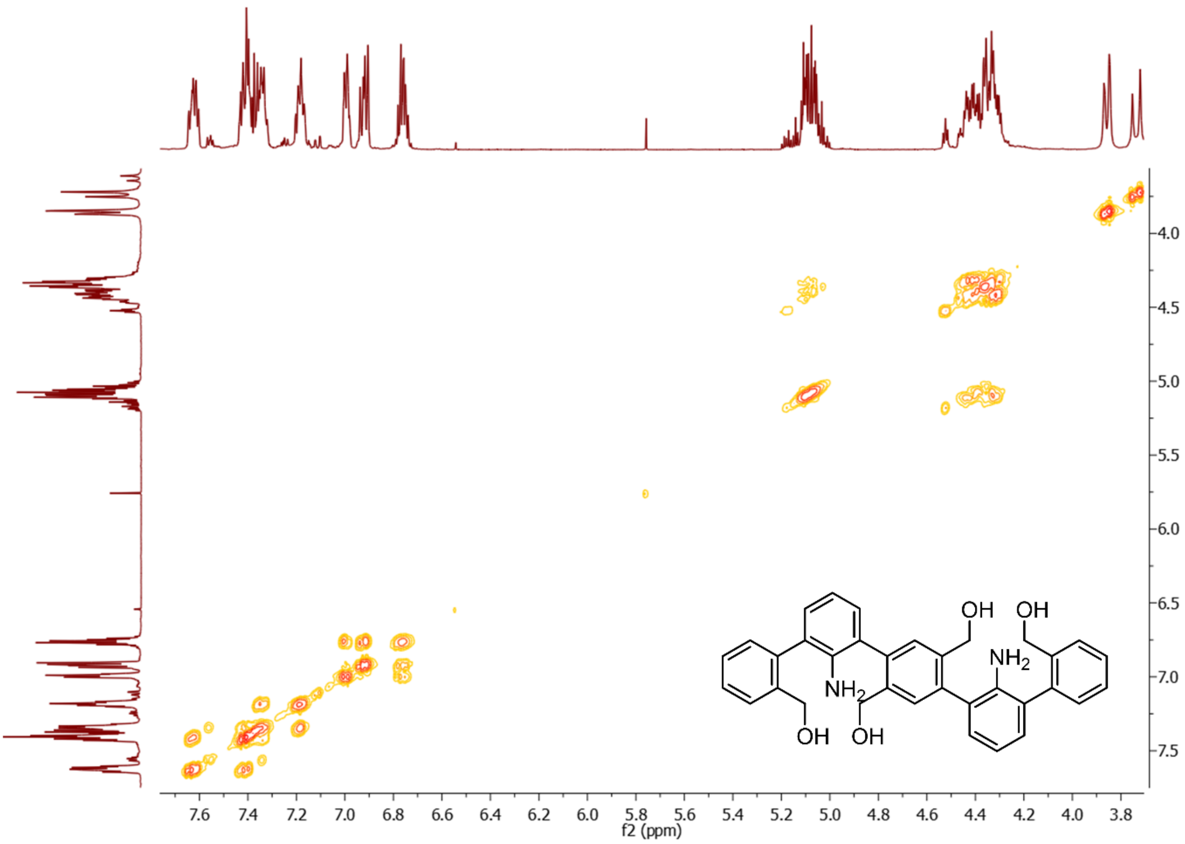

Supplementary Figure 12. COSY spectra of compound **11** at 298 K in DMSO-d_6.

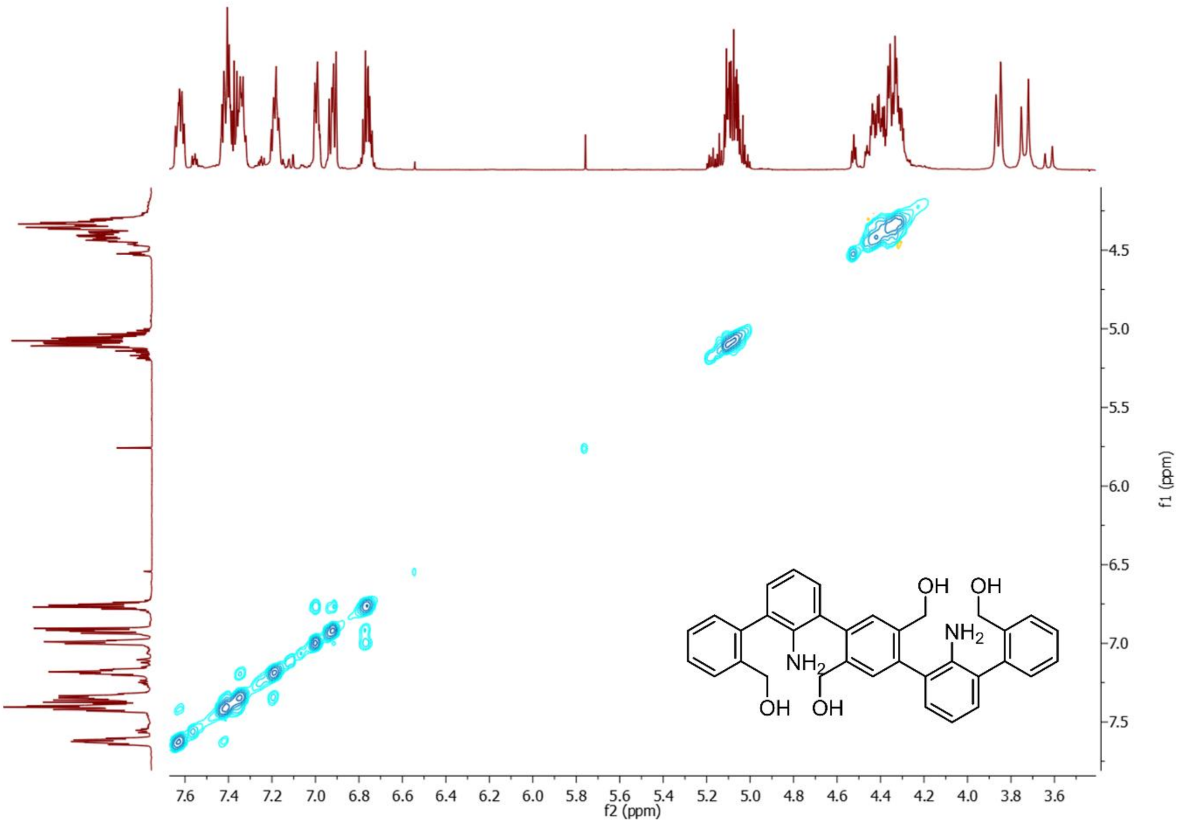

Supplementary Figure 13. NOESY spectra of compound **11** at 298 K in DMSO-d_6.

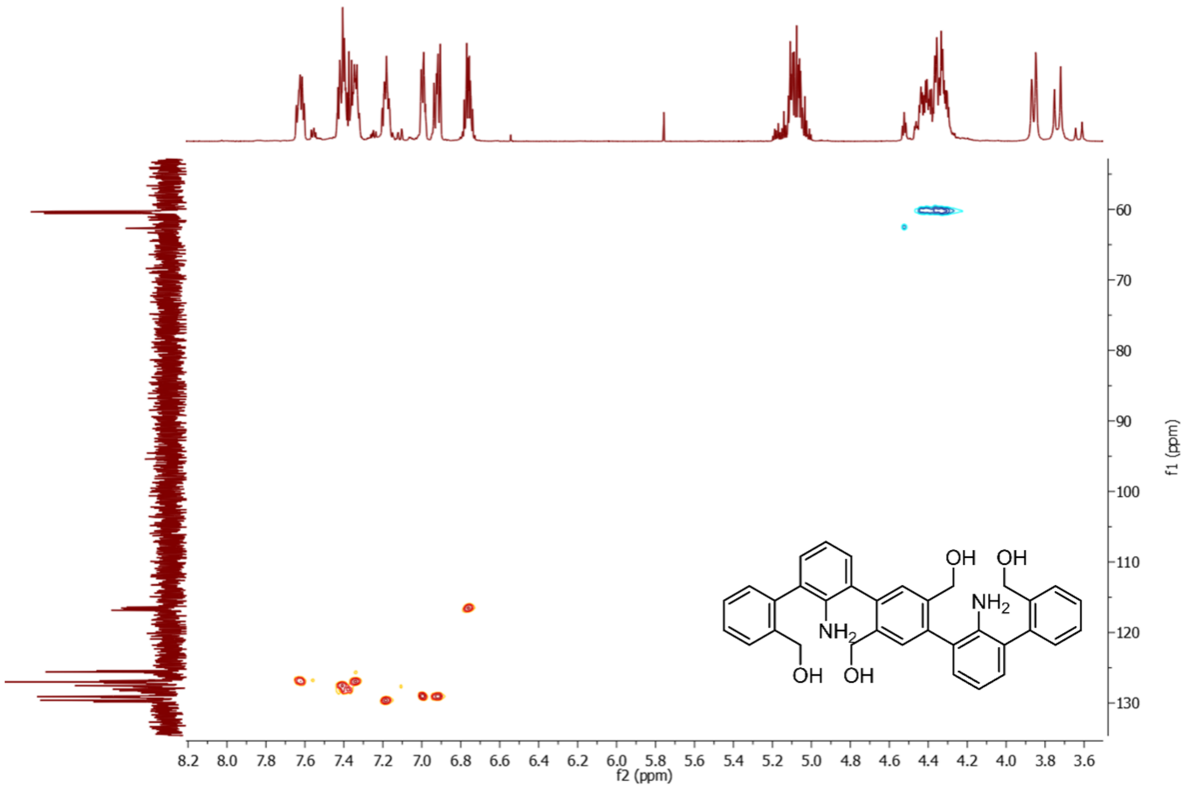

Supplementary Figure 14. HSQC spectra of compound **11** at 298 K in DMSO-d_6.

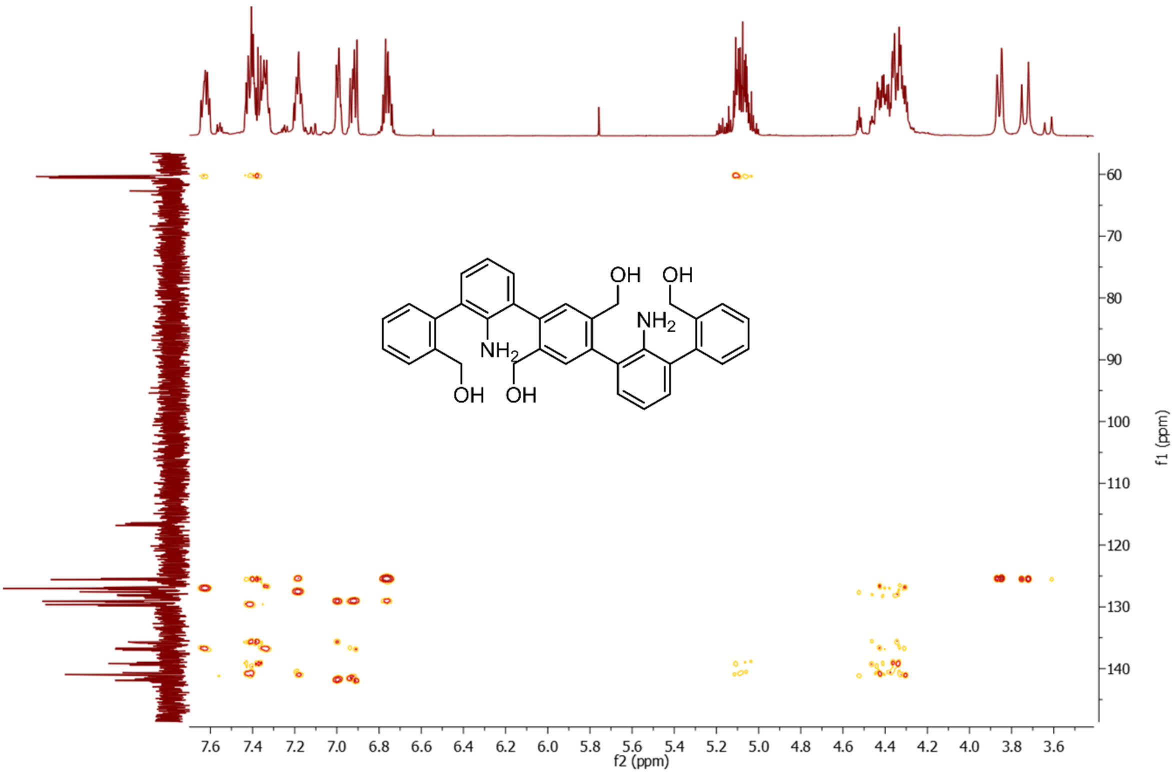

Supplementary Figure 15. HMBC spectra of compound **11** at 298 K in DMSO-d_6.

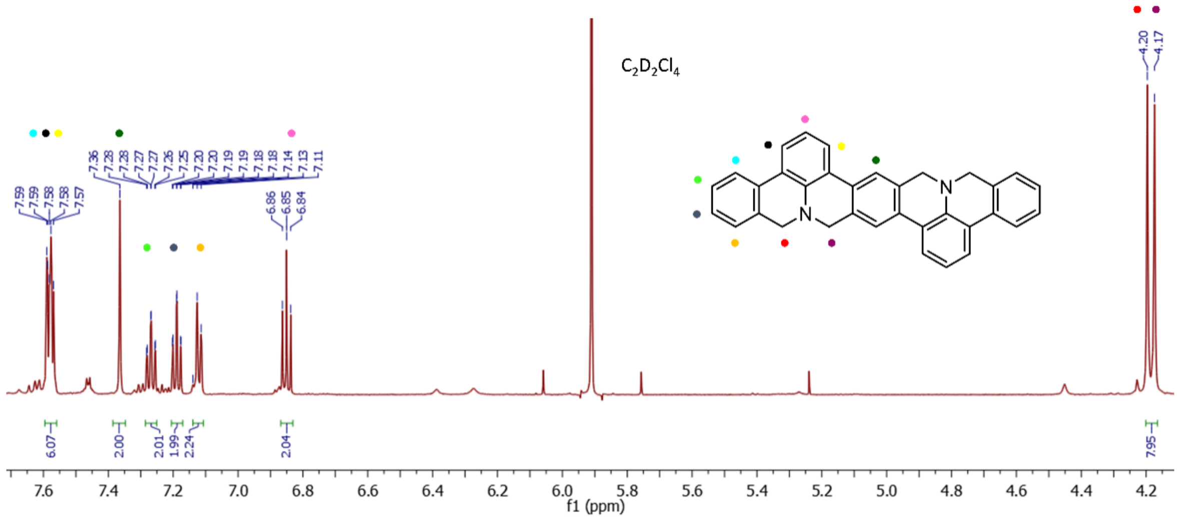

Supplementary Figure 16. ^{1}H-NMR (600 MHz) spectra of compound **1** at 298 K in $C_2D_2Cl_4$.

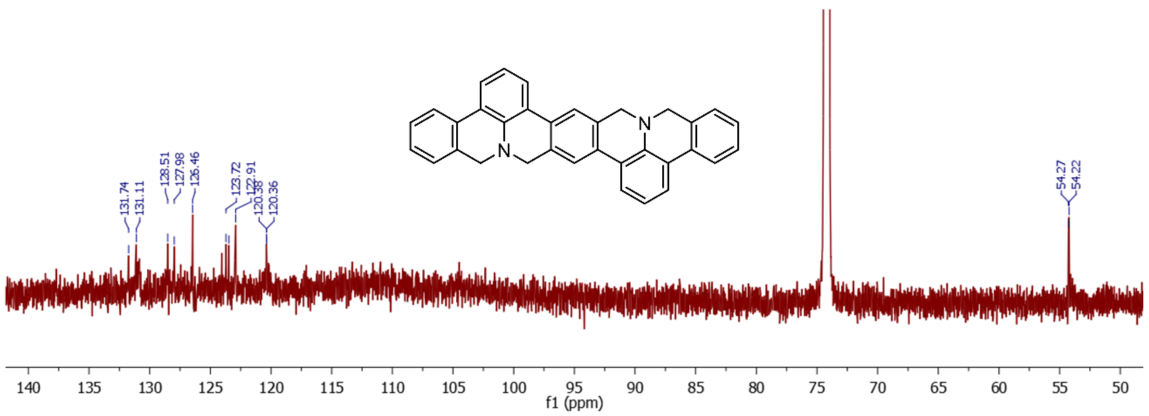

Supplementary Figure 17. ^{13}C-NMR (151 MHz) spectra of compound **1** at 298 K in $C_2D_2Cl_4$.

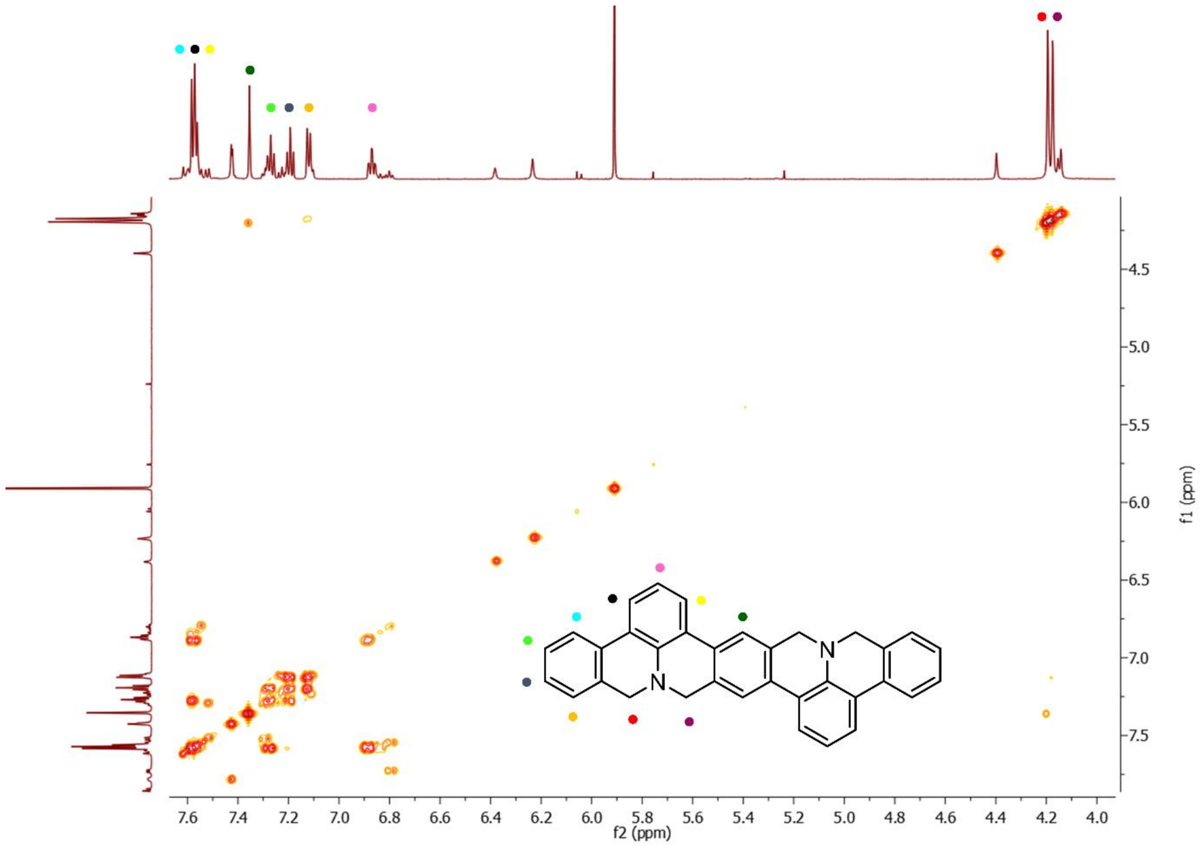

Supplementary Figure 18. COSY spectra of compound **1** at 298 K in $C_2D_2Cl_4$.

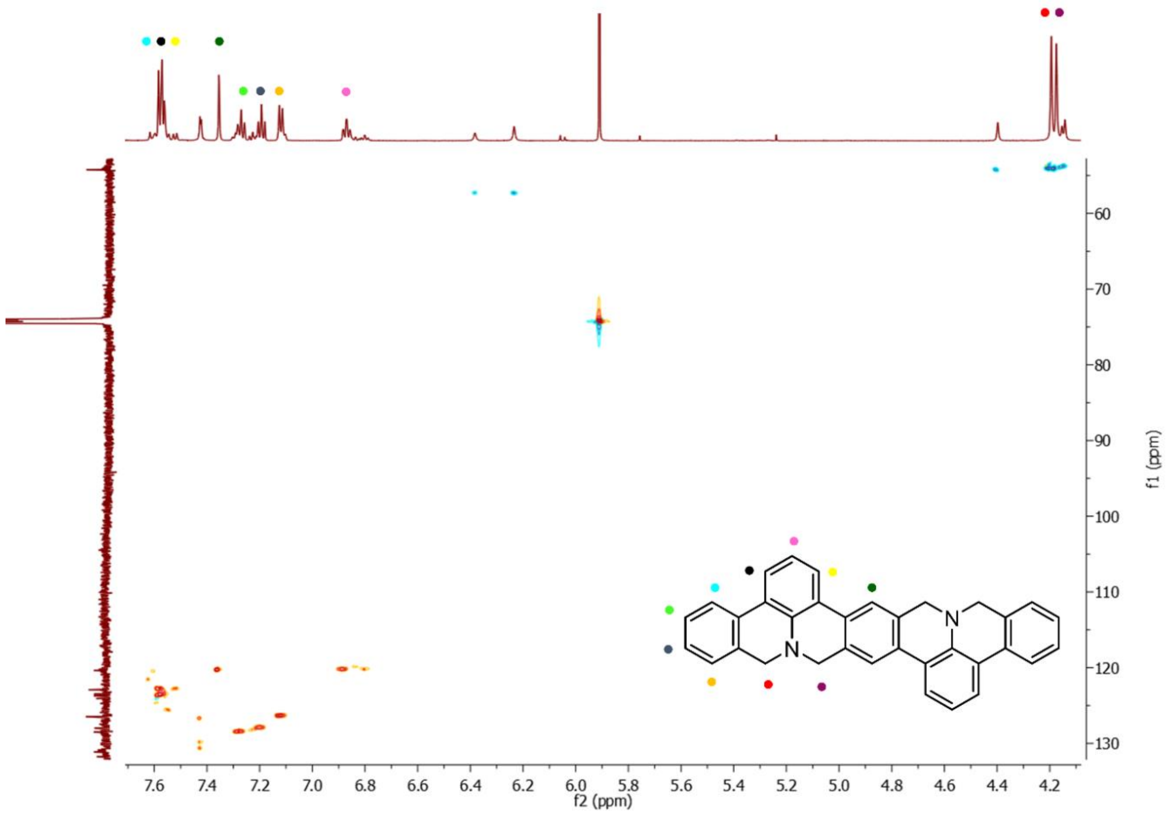

Supplementary Figure 19. HSQC spectra of compound **1** at 298 K in $C_2D_2Cl_4$.

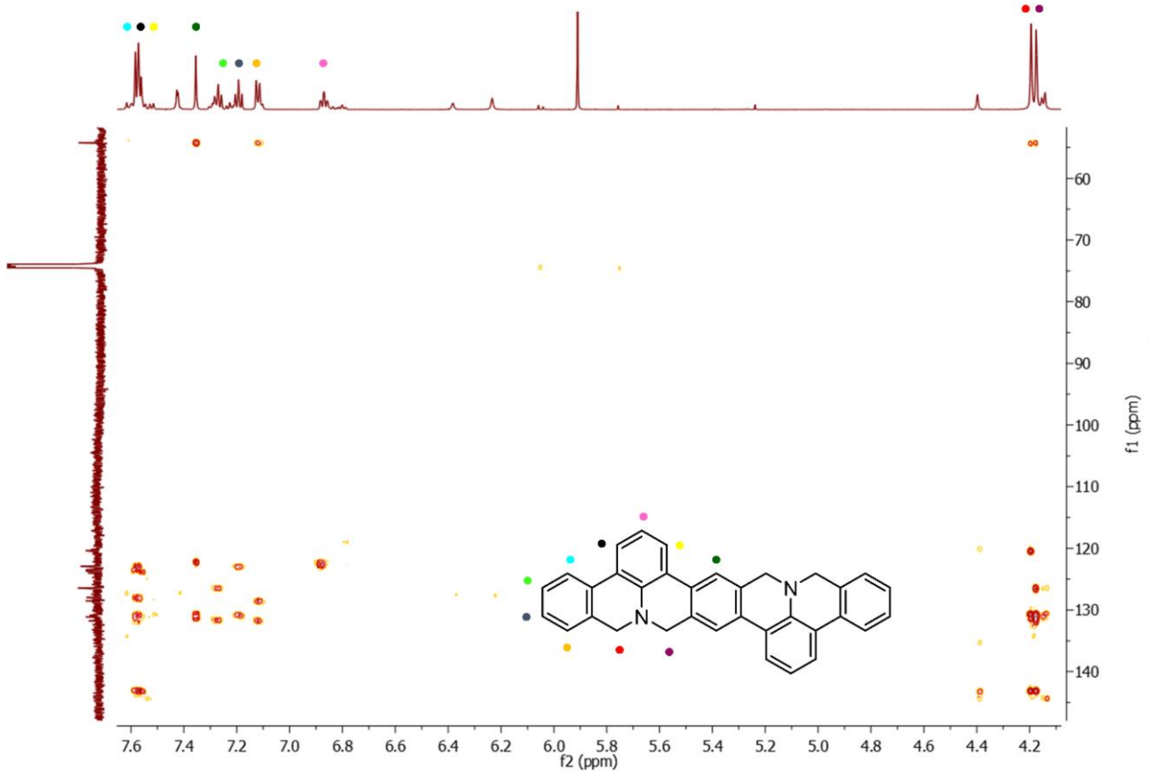

Supplementary Figure 20. HMBC spectra of compound **1** at 298 K in $C_2D_2Cl_4$.

Supplementary Note 5. Infrared Spectroscopy

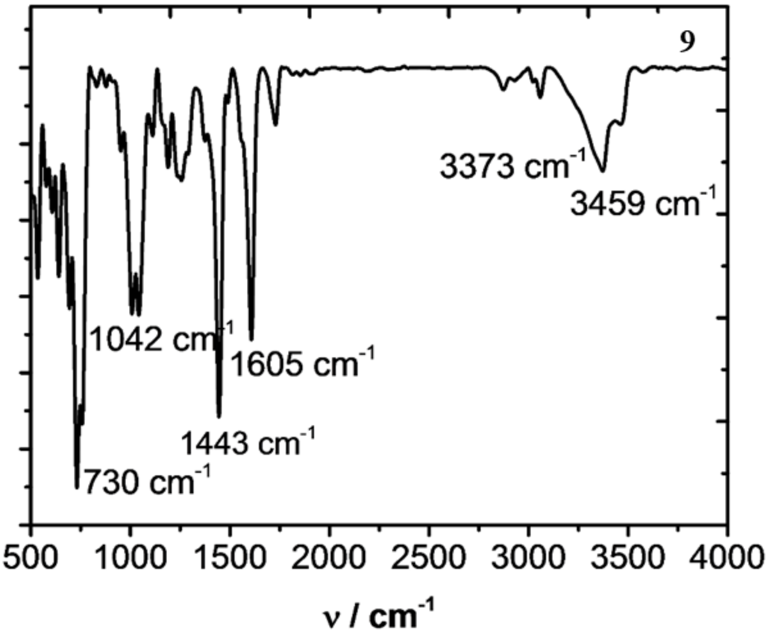

Supplementary Figure 21. IR spectrum of **9**.

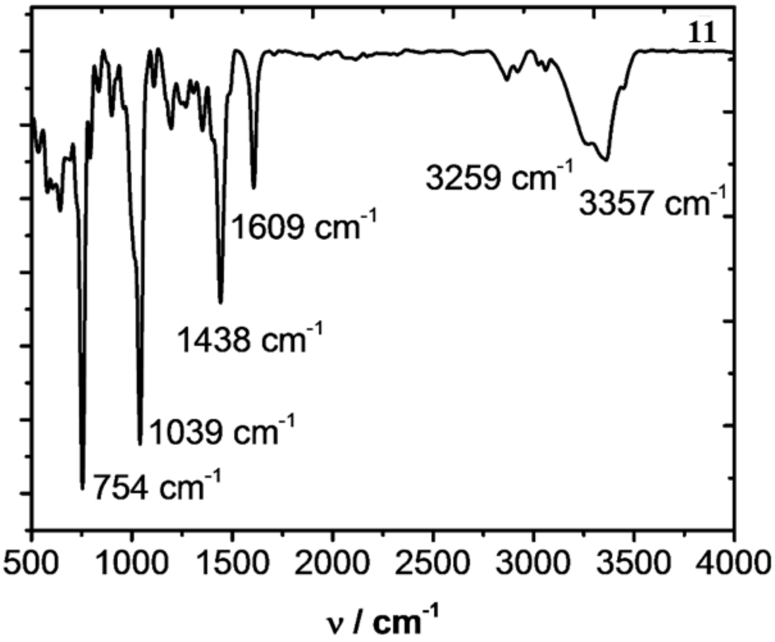

Supplementary Figure 22. IR spectrum of **11**.

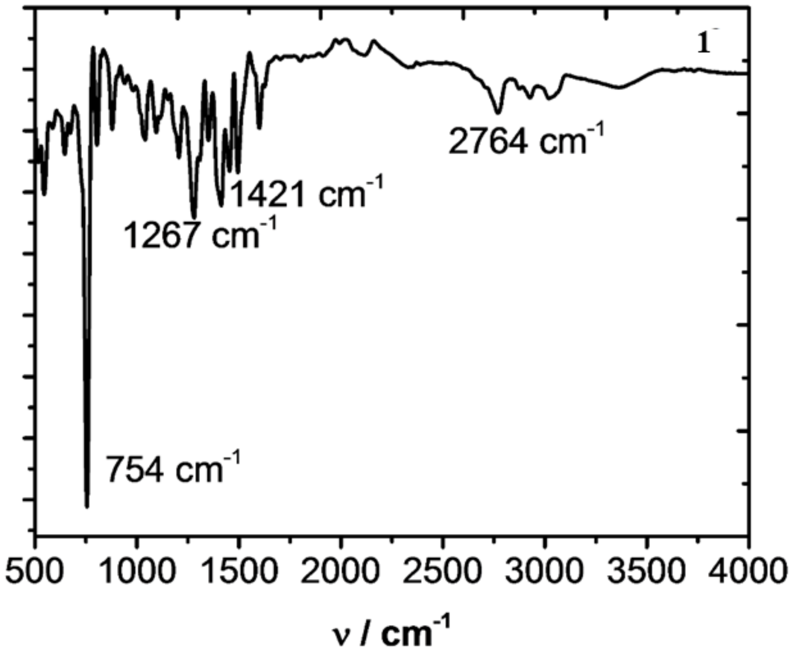

Supplementary Figure 23. IR spectrum of **1**.

■ Supplementary Figures

Supplementary Table 1. Bond length comparison of isolated 3′, 3 and PAMY dimer on Ag(100). PAMY dimer has two major resonance structures, the ionic **3** and the radical **3′**. C(*α*)-N and C(*β*)-N denote the C-N bonds at *α* and *β* sites, respectively.

Types	Bond length (Å)			
	C(*α*)-N	C(*β*)-N	C(*β*)-N	C(*α*)-N
isolated **3′**	1.381	1.388	1.387	1.381
isolated **3**	1.372	1.368	1.368	1.372
PAMY dimer on Ag(100)	1.377	1.366	1.362	1.383

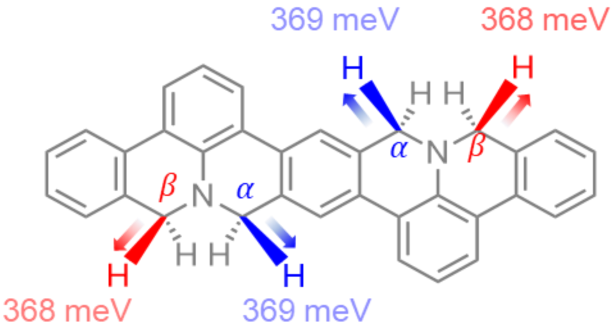

Supplementary Figure 24. Illustration of the specific C-H vibrational modes of **1** in gas phase. Two degenerate phonon modes at 369 meV represent the C-H stretch at *α* site (marked in blue). (b) Two degenerate phonon modes at 368 meV represent the C-H stretch at *β* sites (marked in red).

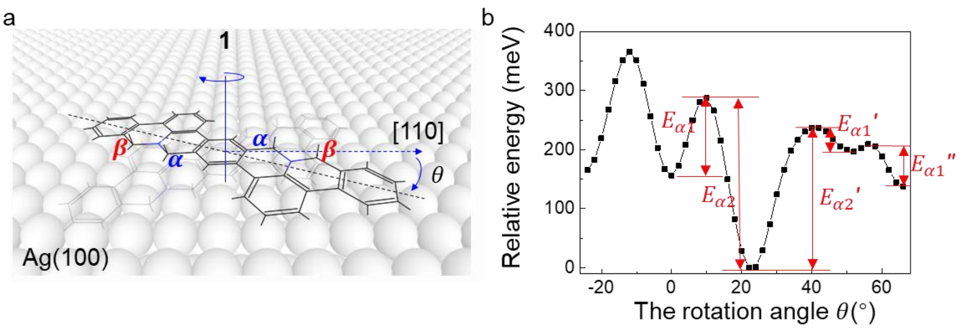

Supplementary Figure 25. DFT Calculated potential energy surface at different rotation angles. (a) Schematics of adsorption configuration of **1** on Ag(100) with different orientations. The long axis of **1**, the [110] direction of Ag(100), and the rotation axis are marked with black dash line, blue dash arrow and blue line. The rotation angle is denoted by θ. (b) DFT calculated potential energy surface at different rotation angles θ. We took 45 possible configurations with each rotation angle differs by 2 degree. In each configuration, the molecule is fully relaxed in z direction. The configurations with rotation angles of 22 and 24 degree exhibit the lowest energies with a difference of 2 meV. Further relaxation finds that the configuration with a rotation angle of 24 degree changes to that with a rotation angle of 22 degree, which is the most possible configuration of molecule 1 on Ag(100).

At 573 K, the diffusion of the molecule can also be activated. The diffusion of the molecule can be divided into two groups. In group I, the molecules diffuse from the other local minimums (configurations with rotation angles of 0, 52 and 66 degree) to the ground state (the one with a rotation angle of 22 degree), as shown in Figure S25; In group II, the molecules diffuse from the ground state to the other local minimums. We define the rate constant of the diffusion from angle 0 degree (52 degree, 66 degree) to the ground state to be $k1$ ($k1'$, $k1''$), and the diffusion from the ground state to the configuration with rotation angle of 0 degree (52 degree, 66 degree) to be $k2$ ($k2'$, $k2''$). Here, we use the energy barrier to replace the activation energy $E\alpha$, as marked in Figure S25. Thus, when T = 573 K,

$$k1 = Ae^{-E_{\alpha1}/RT} = 0.07\,A,\ k1' = Ae^{-E_{\alpha1}'/RT} = 0.44\,A,\ k1'' = Ae^{-E_{\alpha1}'/RT} = 0.24\,A$$

$$k2 = Ae^{-E_{\alpha2}/RT} = 2.96 \times 10^{-3}\,A,\ k2' = k2'' = Ae^{-E_{\alpha2}'/RT} = 8.20 \times 10^{-3}\,A$$

Thus, the population ratio of the ground state to the other local minimum at 573 K is $(k1 + k1' + k1'') : (k2 + k2' + k2'') = 39 : 1$.

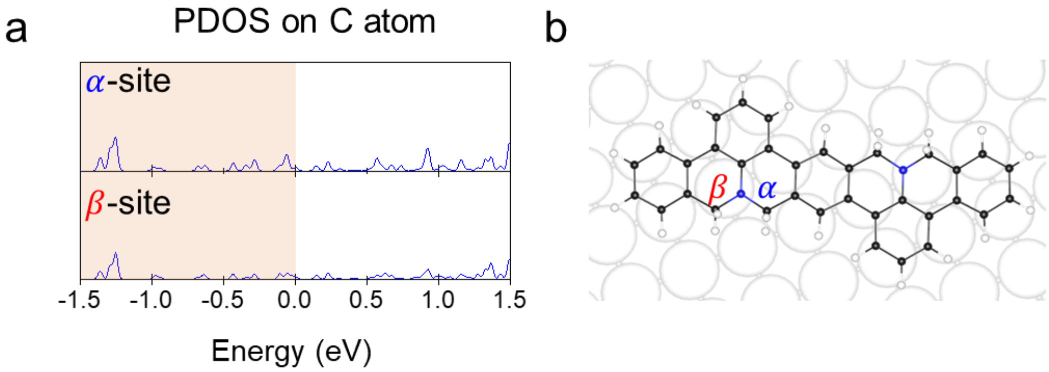

Supplementary Figure 26 (a) Projected density of states (PDOS) on C atoms at α site (upper panel) and β site (lower panel) of molecule **1** on Ag(100). (b) The schematic of molecule **1** on Ag(100).

The $RA_{C(\alpha)/C(\beta)}$ is defined as the ratio of electron density of HOMO contributed from C(α) to that from C(β). Due to the split HOMO peaks after the molecule is adsorbed on substrate (Figure S26 (a)), we obtain the contribution of C(α) or C(β) by integrating the PDOS from the original HOMO to the Fermi level as shown in Figure S26 marked with a light coral background.

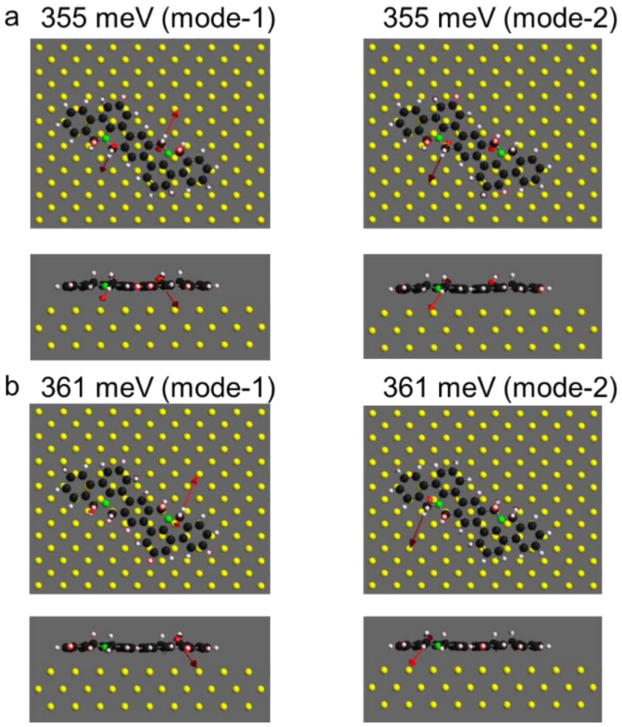

Supplementary Figure 27. DFT calculations of the specific C-H vibrational modes of 1 on Ag(100). (a) Two degenerate phono modes at 355 meV. The two modes represent the C-H stretch at α sites. (b) Two degenerate phono modes at 361 meV. The two modes represent the C-H stretch at *β* sites.

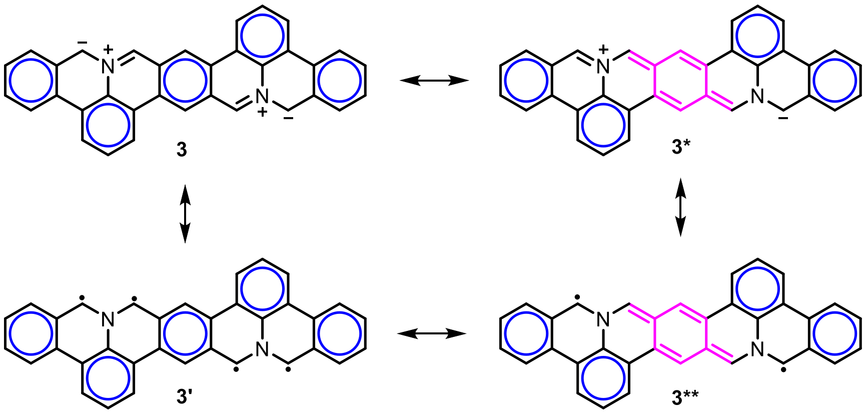

Supplementary Figure 28. Resonance structures of the PAMY dimer.

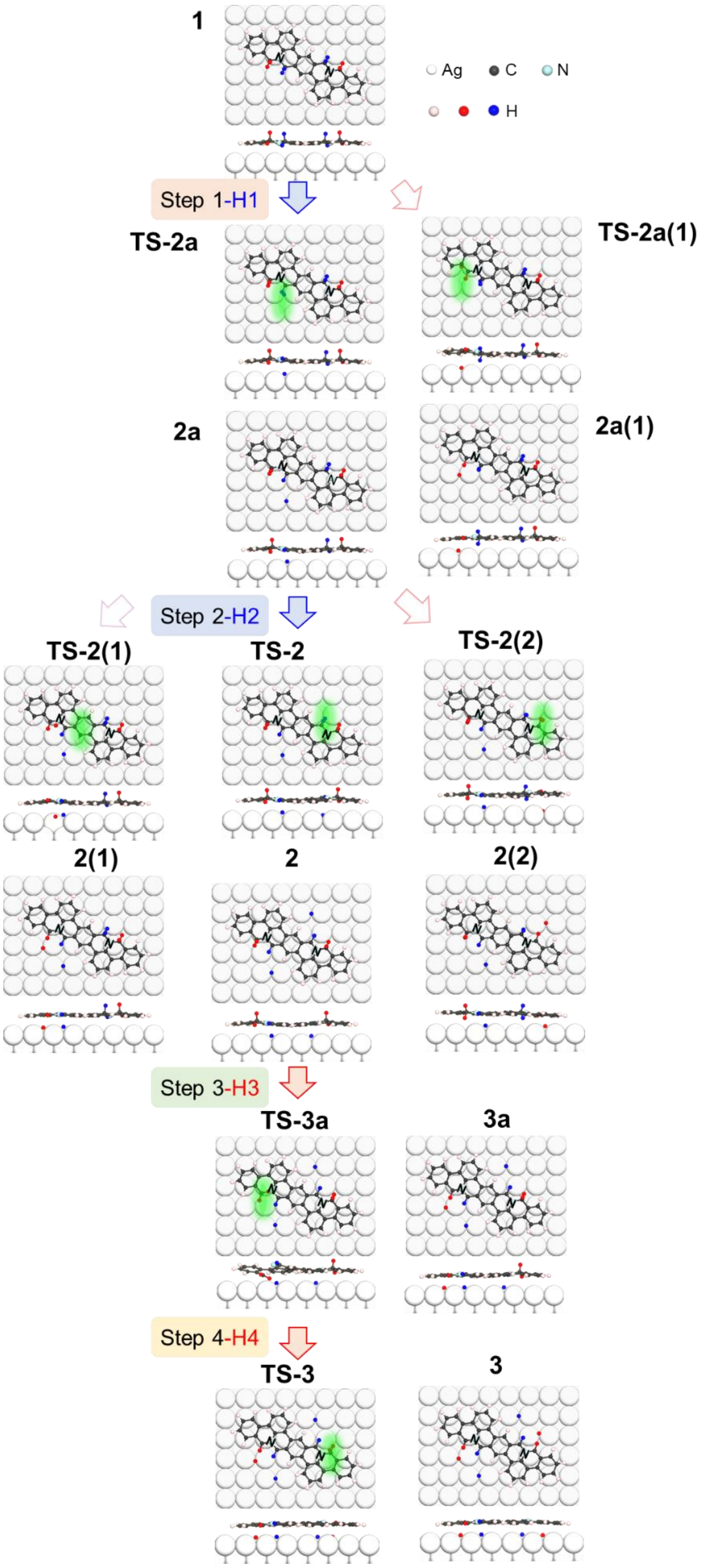

Supplementary Figure 29. DFT calculated structures of initial state, transitional states, metastable states, and final states in Step 1, Step 2, Step 3 and Step 4 in Figure 2.

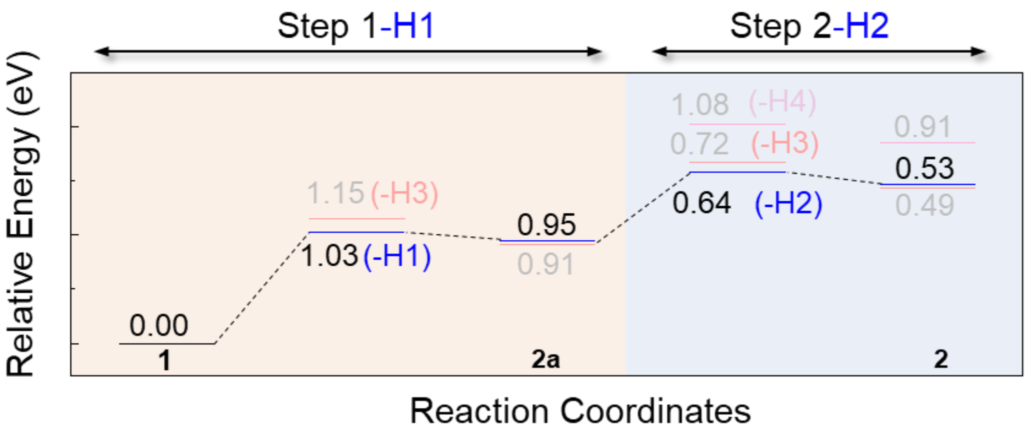

Supplementary Figure 30. DFT calculations on the dehydrogenation barriers of *ortho* C(*sp*^{3}) atoms in the N-heterocycles for molecule 1 on five layers Ag(100) for the first two steps. The energy favourable profile is highlighted while the others are in light colours. According to the energy profile, the dehydrogenation sequence is H1, H2.

We find the calculated energy barrier difference between the removal of H1 and H3 in the first step and that of H2 and H3 in the second step are increased to 0.12 eV and 0.08 eV, respectively, when we consider more layers of substrate.

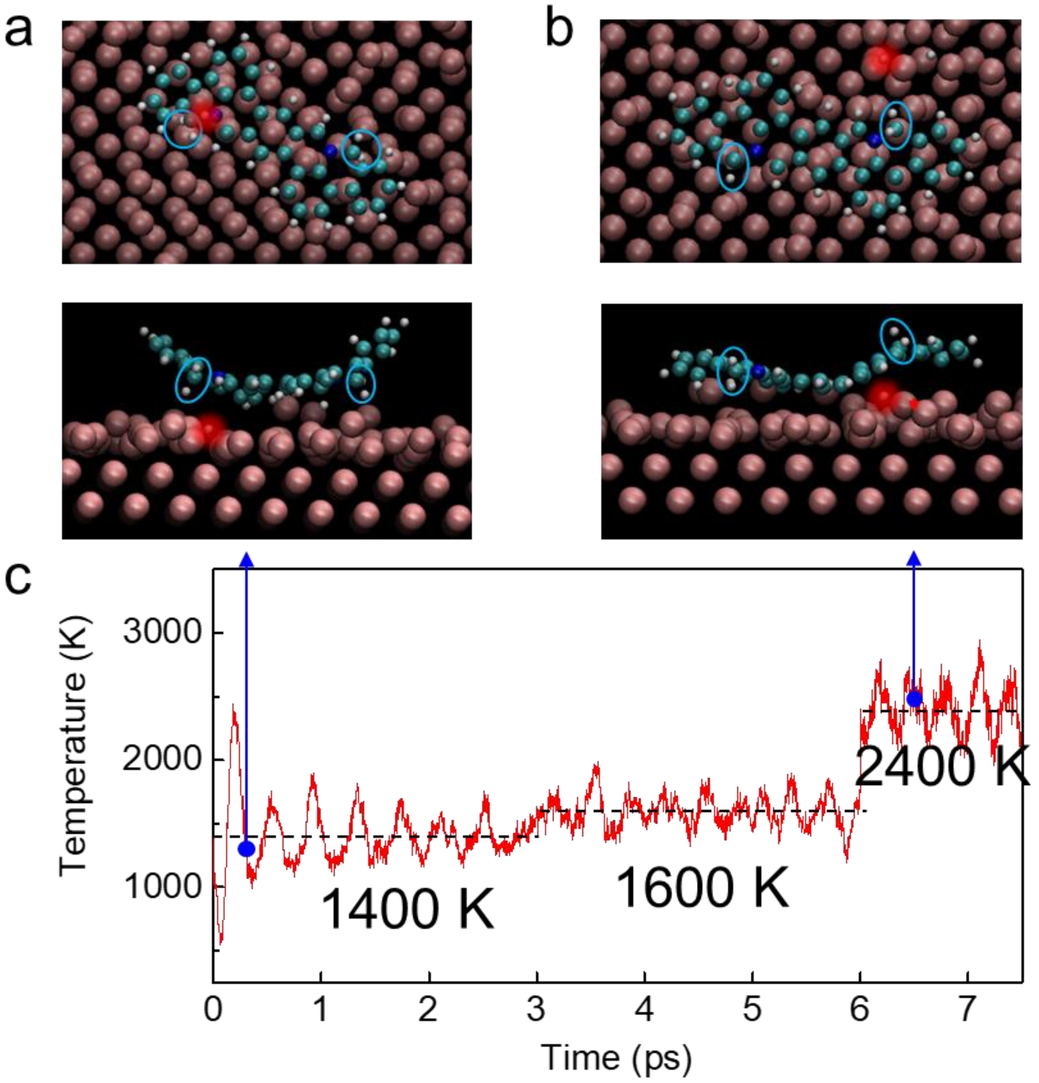

Supplementary Figure 31. Molecular dynamics (MD) simulation of precursor (1) at 1400 K for 3 ps, 1600 K for 3 ps, 2400 K for 1.5 ps. (a) Top and side views of the snap shot at 1400 K. The H at α site (H1, marked with red shadow) detached first, while the other H atoms still bond with C atoms. The H atoms at β sites are marked in light blue circles. (b) Top and side views of the snap shot at 2400 K. The H at the other α site (H2, marked with red shadow) detached, while the other H atoms still bond with C atoms. (c) Temperature variation with time, and the bule dots denote the times of snap shots taken in (a-b).

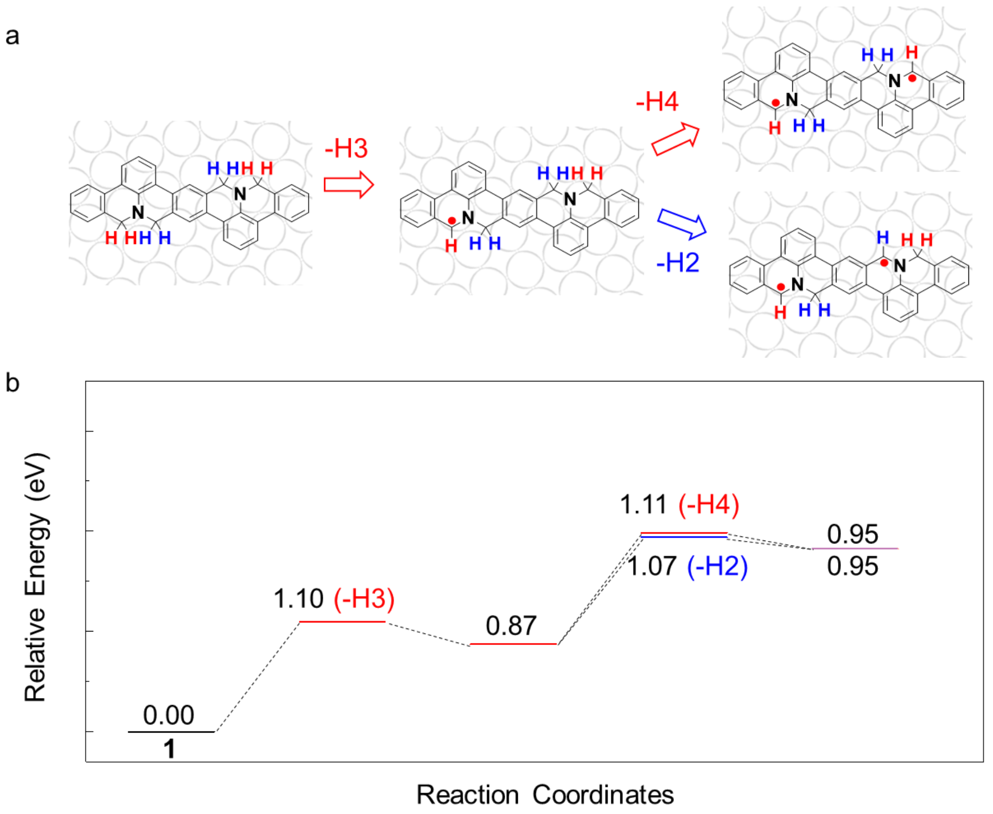

Supplementary Figure 32. DFT calculations on dehydrogenation barriers of the two *ortho* C(sp^3) atoms in the N-heterocycles in 1 on Ag(100). (a) Schematics of initial state (1), metastable state and final state of two dehydrogenation processes (H3 first and then H4, H3 first and then H2). (b) DFT-calculated energy profiles along the two dehydrogenation paths. The energy barriers are higher than the dehydrogenation sequence of H1 first and then H2, which strengthen our statement that the dehydrogenation sequence of H1, H2, H3 and H4 is favored.

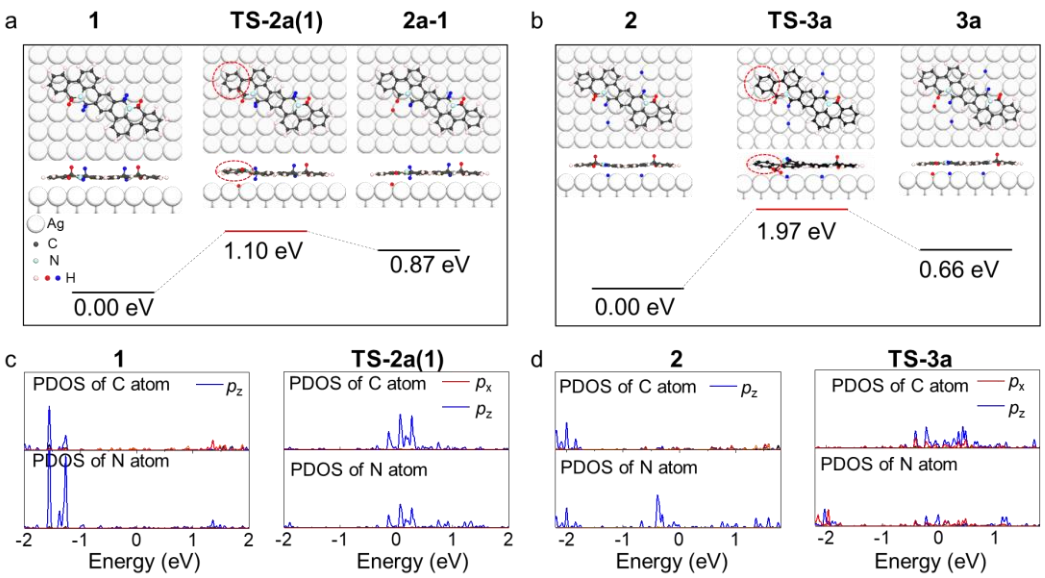

Supplementary Figure 33. (a, b) Atomic structures (top panel) and relative energies (bottom panel) of initial states, transition states and final states in two dehydrogenation processes involving H3 atoms. (c, d) Calculated PDOS onto the C(*β*) and adjacent N atoms in initial states **1**, **2** and transition states **TS-2a(1)**, **TS-3a**. The white, black and light blue balls represent Ag, carbon and nitrogen atoms, respectively. The hydrogen atoms bond with C(*β*) and C(alpha) are colored in red and blue, respectively. Other hydrogen atoms are colored in light pink.

During two dehydrogenation processes, the adjacent benzene rings are both distorted (marked by the red dashed circles in Figure S33(a-b)), which decreases the planarity of conjugated molecules. The out-of-plane buckling of **TS-3a** is larger than that of **TS-2a(1)**. The decrease of planarity of **TS-3a** compared with **TS-2a(1)** leads to a higher barrier due to a less conjugated transition state. Meanwhile, we analyzed the PDOS onto the C(*β*) and adjacent N atoms in the initial states and transition states of the two processes. The results show both p_x and p_z orbitals contribute to the PDOS in C and N atoms in transition state **TS-3a**, while only p_z orbital contributes to that in **TS-2a(1)**, as shown in Figure S33 (c-d), which further confirms the decrease of conjugation of **TS-3a**. Therefore, we conclude that the decrease of conjugation of **TS-3a** leads to a higher barrier in the pathway starting from metastable state **2** than that from the initial state **1**.

To understand the role of substrate in the dehydrogenation process, we calculated the dehydrogenation barriers of an isolated molecule **1** in the gas phase. The **1** is buckled in gas phase (Figure S6a) while it becomes flat after adsorption on Ag(100). Our calculations of **1** at gas phase show that the dehydrogenation barrier of the two H atoms (H1 and H2) at α sites is 3.32 eV, and the reaction path is shown in Figure S5. Considering the much lower dehydrogenation barriers on surface than those in gas phase, Ag(100) surface plays an important role in the dehydrogenation process of **1**.

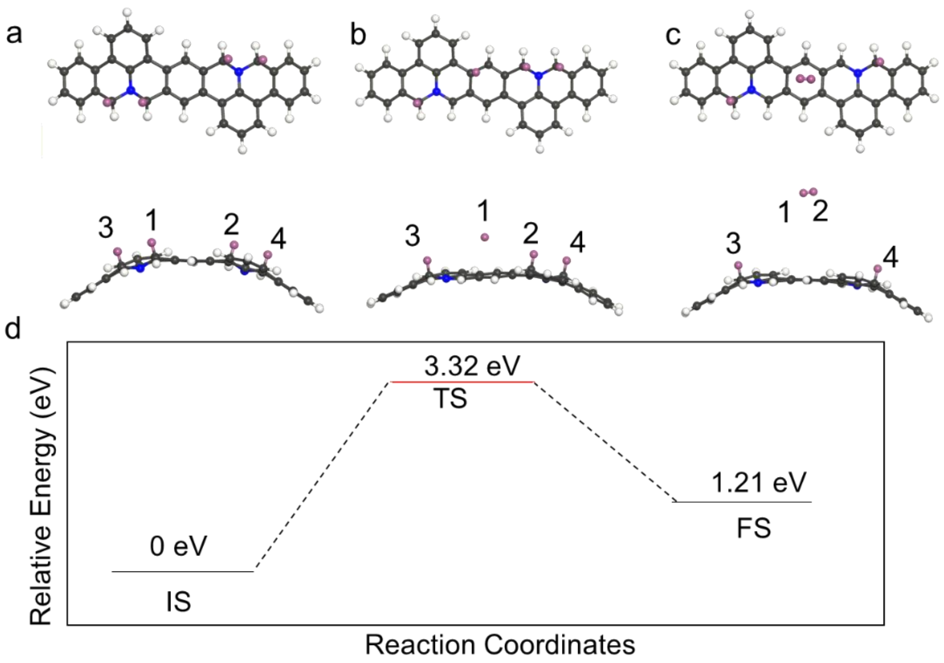

Supplementary Figure 34. **DFT calculated pathway of the hierarchical dehydrogenation reactions at H1 and H2 sites in gas phase.** (a)-(c) Top and side views of local minima (IS and FS) and transition states (TS) along the reaction path. (d) Corresponding energy profiles.

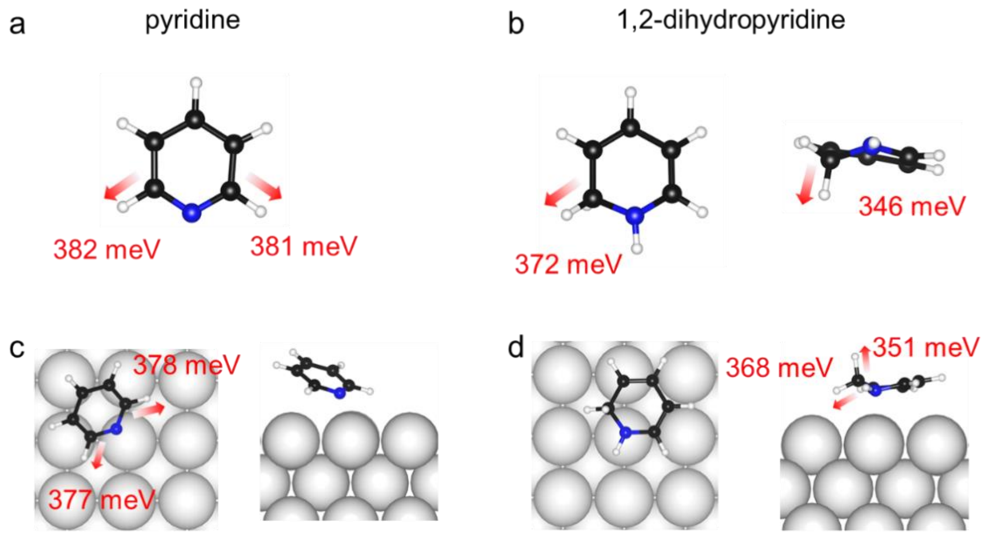

Supplementary Figure 35. Illustration of *ortho* C-H vibrational modes of pyridine and 1,2-dihydropyridine. (a) and (c) Two phonon modes of *ortho* C(sp^2)-H stretch in isolated pyridine and after pyridine adsorption on Ag(100). (b) and (d) Two phonon modes of *ortho* C(sp^3)-H stretch in isolated 1,2-dihydropyridine and after 1,2-dihydropyridine adsorption on Ag(100).

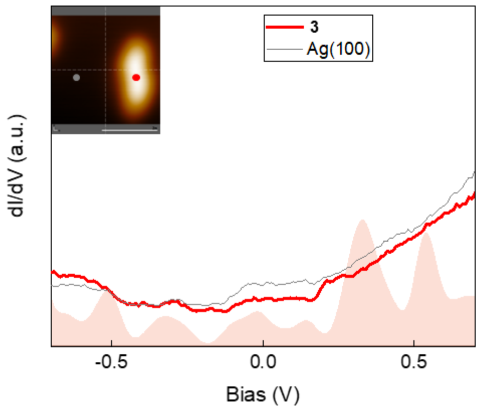

Supplementary Figure 36. Experimental dI/dV mapping on 3. Red and gray lines are STS on **3** and on Ag(100), respectively. The inset is the STM image of a 3 on Ag(100). The scale bar is 2 nm. The red and grey dots indicate the positions we obtain the STS. The light coral shadow is calculated projected density of states (PDOS) of **3** on Ag(100). The STS follows the calculated DOS spectral shape of **3** on Ag(100) (the light coral shadow).

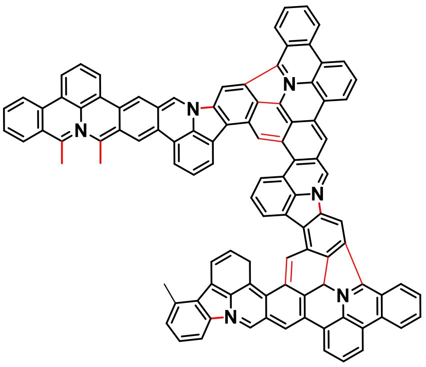

Supplementary Figure 37. The intermolecular coupling could be induced by **3** with homochirality.

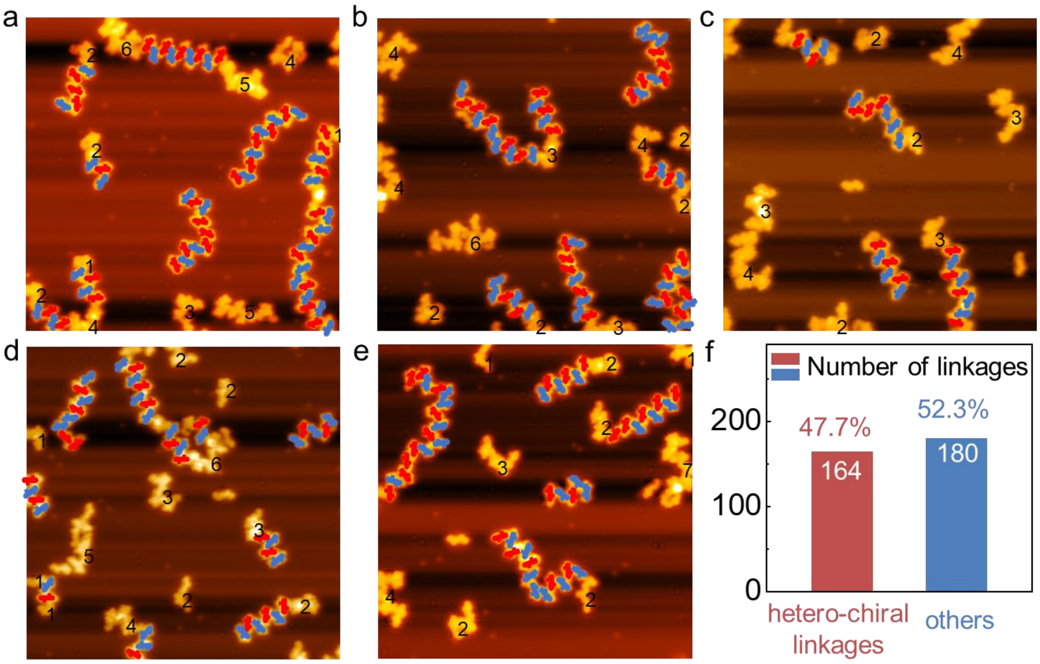

Supplementary Figure 38. (a-e) STM images (20 nm × 20 nm) of ribbons acquired with a CO functionalized tip. The W-shape ribbon **5** is constructed by enantiomers with different chirality. We highlight two enantiomers with different chirality using blue and red shadows. (f) Statistics on the population of hetero-chiral linkages and other linkages.

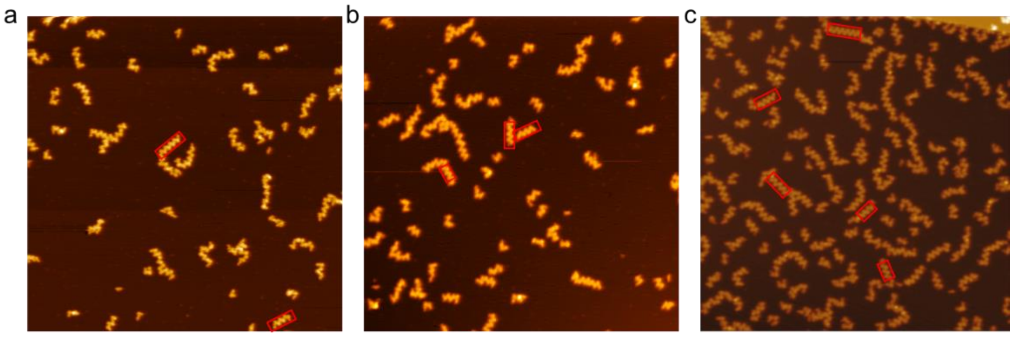

Supplementary Figure 39. (a-c) Large-scale (100 nm × 100 nm) STM images of the ribbons. The red boxes highlight the W-shaped ribbons that consist of more than 4 monomers.

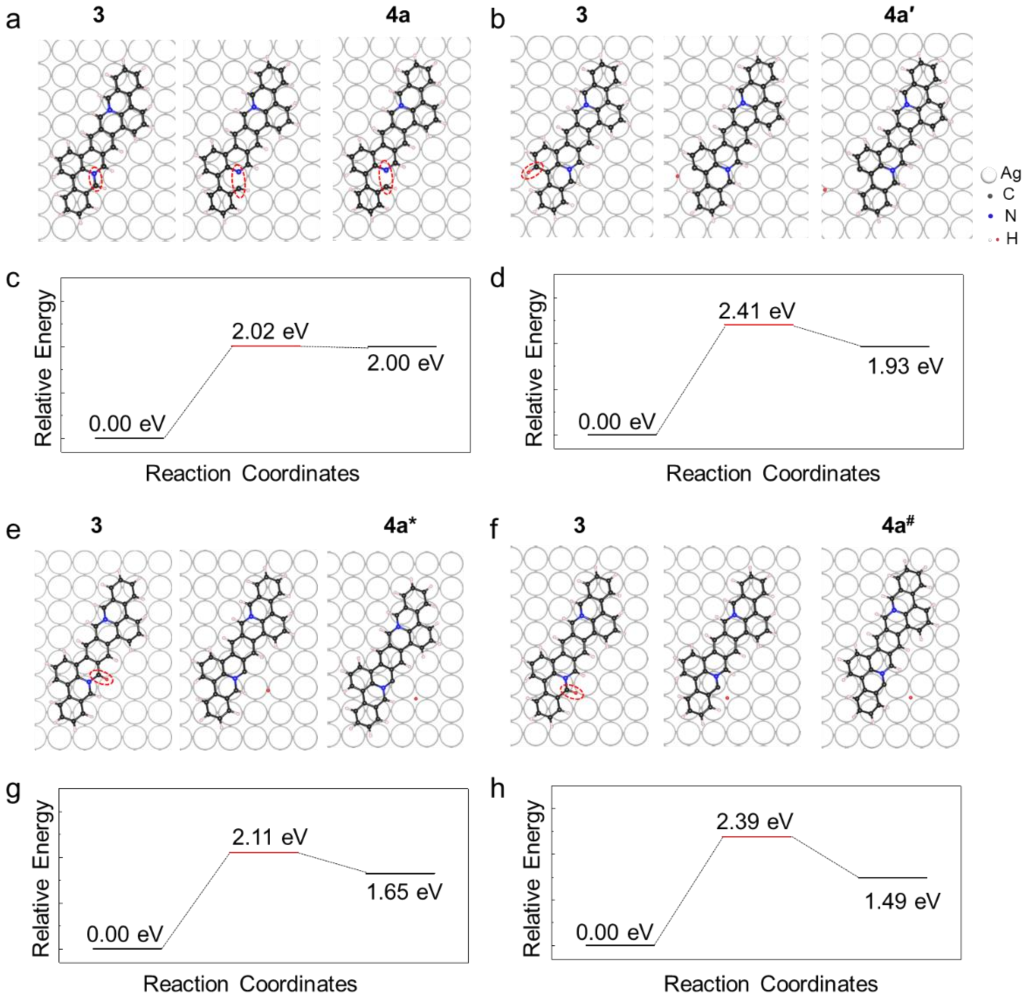

Supplementary Figure 40. DFT calculations on cleavage energy barriers of C-N and C-H bond in 3 on Ag(100). (a-b, e-f) Atomic structures of initial states, transition states and final states in C-N and C-H cleavage processes. (c-d, g-h) DFT-calculated energy profiles along four paths. The white, black and blue balls represent Ag, carbon and nitrogen atoms, respectively. The detached hydrogen atom is colored red. Other hydrogen atoms are colored in light pink.

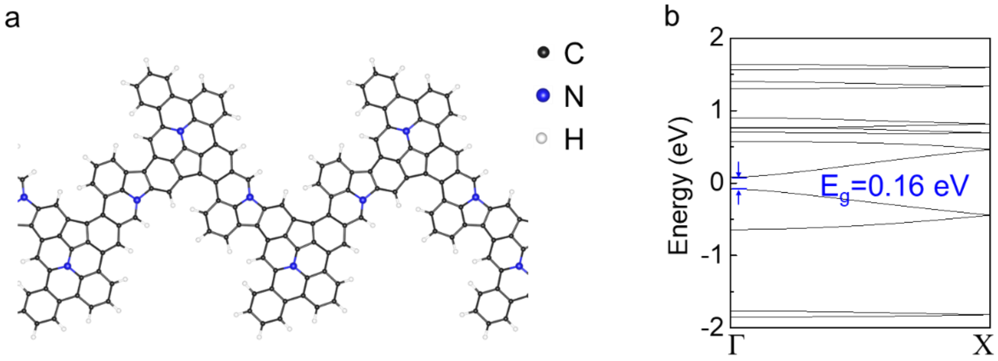

Supplementary Figure 41. (a) Atomic structure and (b) band structure of the W-shape ribbon **5**.

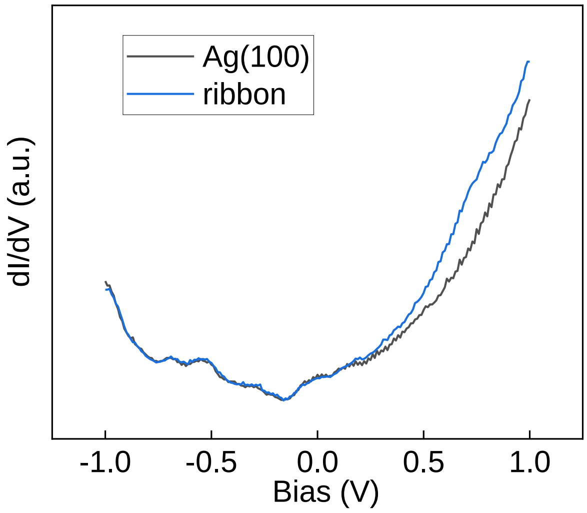

Supplementary Figure 42. Experimental dI/dV mapping on the ribbon on Ag(100) substrate.

■ Supplementary References

1. Berger, R.; Giannakopoulos, A.; Ravat, P.; Wagner, M.; Beljonne, D.; Feng, X.; Müllen, K. *Angewandte Chemie International Edition* 2014, 53, 10520-10524; *Angewandte Chemie* 2014, 126, 10688-10692.
